# Embracing chaos: the unpredictability of animated logos shapes users’ sustained attention

**DOI:** 10.3389/fpsyg.2025.1642722

**Published:** 2025-08-06

**Authors:** Wen Guan, Dong Min Cho, Li Zheng

**Affiliations:** ^1^Department of Design and Manufacturing Engineering, Jeonbuk National University, Jeonju, Republic of Korea; ^2^Department of Industrial Design, Jeonbuk National University, Jeonju, Republic of Korea; ^3^Department of Business, Qingdao University, Qingdao, Shandong, China

**Keywords:** animated logos, perceived unpredictability, perceived novelty, brand types, sustained attention, eye-tracking

## Abstract

**Introduction:**

Animated logos have become a substantial investment in brand marketing, and designers tend to use flashy presentation effects for brand animations. Brands must understand how effectively animated logos capture users’ attention in media communication of visual distractions. However, the reasons for the impact on users’ sustained attention are not yet clear. By investigating users’ perceptions of animated brand logos, this study proposes a new dimension: “perceived unpredictability.”

**Methods:**

Combining previous studies on physics, psychology, and marketing, we analyze 63 actual cases and demonstrate an inverted U-shaped relationship between perceived unpredictability and sustained attention. Study 1 conducted a moderated mediation analysis using 1,844 questionnaires.

**Results:**

There exists a significant mediating effect through users’ perceived novelty, while brand type acts as a moderator in this process. Specifically, animated logos with unpredictable features in feel brands with hedonic emotional appeals enhance users’ sustained attention, while the opposite is true for think brands with utilitarian cognitive appeals. To avoid confounding effects of actual cases, Study 2 partially replicated the findings regarding the effect of perceived unpredictability on sustained attention under more demanding conditions using eye-tracking, further confirming the robustness of the results.

**Discussion:**

Our study provides new evidence for mechanisms by which animated logos affect sustained attention through self-report and behavioral measures and reveals which brands can benefit from animated logos with unpredictable characteristics. These findings provide feasible suggestions for marketers and designers to enhance brand influence.

## Graphical abstract

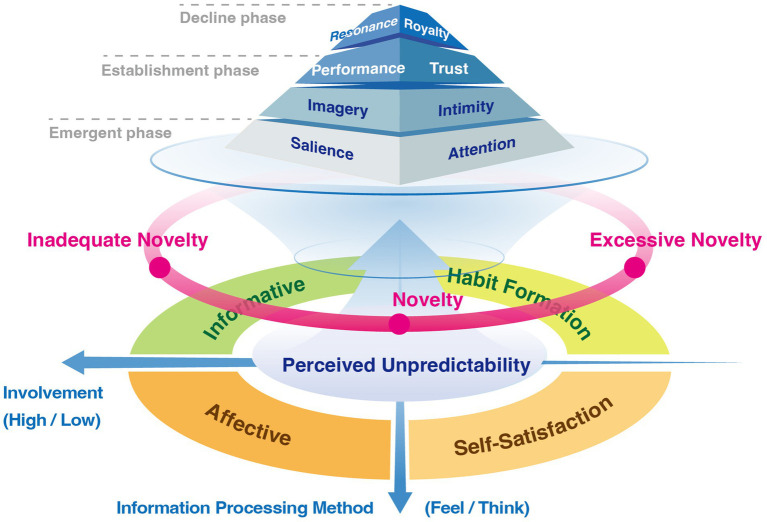

## 1 Introduction

“Nothing happens until something moves.” In urban environments full of digital screens, animated logos have emerged as a powerful medium for brand communication. Despite the visual noise around them, some logos still stand out. What makes certain animated logos grab attention while others are ineffective? These questions lie at the heart of animated logo design research. Some researchers argue that well-designed animated images significantly enhance users’ attention to brands and their willingness to purchase (Lang et al., 2002; Laroche et al., 2022; Su, 2024). Prior studies have demonstrated that movement factors may prompt consumers’ higher-level judgments, subsequently impacting decision evaluation or behavior (Baxter and Ilicic, 2018; Peng et al., 2023). Others contend that animated images are ignored in visual overload (Khacharem et al., 2015; Yoo and Kim, 2005). Previous research on animated advertising suggests that distraction is a key limitation, as animation can interfere with viewers’ cognitive processing of the advertising message (Yoo and Kim, 2005; Goldstein et al., 2014). How can the motion principles of animated logos enhance the effectiveness of digital brand communication? That is the focus of this article. In the early stages of brand development, capturing users’ sustained attention is a critical first step toward increasing brand salience and accumulating brand assets (Keller, 1993). This article aims to identify which motion principles are most effective in capturing user visual attention. Addressing this question is vital for emerging brands seeking to enhance their visibility. We also care about the degree of matching between animated logos and brand types. Animated logos that match the brand cues are more effective in brand communication, while incompatible animated logos may weaken brand recall (Brasel and Hagtvedt, 2016). Therefore, this study will focus on the motion principles of animated logos and explore their impact on brand communication.

Motion principles are a key step in transitioning from static to dynamic design. However, their application in animated logos is still underexplored. From an academic perspective, existing research focuses on the impact of brand logos’ implied motion on user behavior. That is fundamentally different from the animated logos that move on the screen (Cian et al., 2015; Baxter and Ilicic, 2018; Luffarelli et al., 2019; Wang et al., 2023). From the perspective of market practice, designers rely on their own experience when creating animation and lack a set of animation design bases for novice designers. Therefore, our study introduced the relevant theories of physics (motion principles based on energy conservation), cognitive psychology (cognitive load theory, cognitive dissonance theory, and attention selection), and marketing to support it. This paper explores how, why, and when animated logos affect users’ perceptions and analyzes the moderating influence on perceived novelty and sustained attention from the perspective of brand type heterogeneity.

Our study differs from the existing literature on animated logos in three ways. First, we incorporated physics-based concepts into brand marketing and proposed a new dimension in animated logo design: “Perceived Unpredictability.” Previous studies have proposed the positive influence of perceived animacy on brand communication (Peng et al., 2023), we are interested in exploring more dimensions that have a positive influence on brand communication effectiveness. Second, we found a nonlinear relationship between perceived unpredictability and sustained attention. Cognitive dissonance theory and cognitive load theory provide theoretical support for our hypothesis. Third, we explore the influence of perceived novelty and brand type consistency in the marketing process. We find that feel brands are positively associated with perceived unpredictability, while the opposite is true for think brands.

Based on the above speculation, we conducted two studies. Study 1 evaluated the impact of perceived unpredictability on users’ sustained attention through 63 actual cases of animated logos. We also conducted a mediating regression analysis of perceived unpredictability (independent variable), perceived novelty (mediator variable), and users’ sustained attention (dependent variable). A controlled experiment conducts the moderating effect of brand types in this process. Study 2 aimed to replicate the findings of Study 1 under more stringent conditions. Finally, we conducted interviews to record users’ brand preferences, exploring the marketing effectiveness of animated logos.

Our study provides several theoretical and practical contributions to animation design and brand marketing. Specifically, we provide evidence that perceived unpredictability can significantly impact users’ sustained attention, thus identifying the effective influence of animated logos in brand marketing. Our study also emphasizes the importance of specific motion principles (including speed, amplitude, direction, and structural complexity) in shaping this effect. Furthermore, we found that perceived novelty is a hidden pathway influencing sustained attention. In other words, perceived novelty can serve as a basis for judgment to limit the adverse impact of perceived unpredictability on sustained attention. We also observe the effectiveness of the motion principles under different brand types with brand marketing. In summary, our study explores the role of animated logos in brand marketing and reveals previously unexplored dimensions. The final chapter discusses these contributions in detail.

## 2 Theoretical review and hypothesis

### 2.1 Animated logos and motion principles

#### 2.1.1 Animated logos

The widespread adoption of mobile devices has created fertile ground for disseminating animated logos. However, for companies interested in using them, it’s essential to understand whether such logos can generate measurable benefits (Guido et al., 2016). Marketing research shows that static visual design elements such as logo color, size, form, and orientation can influence consumer perceptions and shape subsequent decision-making (Pieters and Wedel, 2004; Zhang et al., 2006; Hagtvedt, 2011; Park et al., 2013; Miceli et al., 2014; Bresciani and Del Ponte, 2017; Huang et al., 2021; Xie et al., 2021; Peng et al., 2022). As digital brands evolve, scholars discuss the differences in the impact of static and animated design on brand communication (Bruce et al., 2017; Huang et al., 2021). Research shows that animated presentations are more effective than static ones in enhancing consumer evaluations and behavioral intentions (Kim and Lakshmanan, 2015; Roggeveen et al., 2015; Mourey and Elder, 2019; Peng et al., 2023). Most existing studies focus on implied motion in static graphics. Cian et al. (2015) examined how subtle variations in dynamic tension within warning sign icons influence human behavioral responses. Baxter and Ilicic (2018) systematically examined how the “force” in dynamic logos shapes brand attitudes and purchase intentions. Academics usually use the terms “dynamic” and “animation” to distinguish this essential difference. In the era of digital design, to make brands stand out in the market, “animated logos” that can move on the screen have received more attention. Therefore, our study explores the impact of animated logos on user attention mechanisms.

#### 2.1.2 Motion principles in animated logos

The concept of motion principles originated in physics, which describes the states governing the movement of objects, including physical properties such as velocity, acceleration, and direction. Research on motion principles in animated logos has primarily focused on the dimension of perceived vitality. Variables such as motion speed (Pratt et al., 2010), direction (Tremoulet and Feldman, 2000; Kawabe, 2017), and expansion or contraction of graphics (Parovel et al., 2018) have been commonly employed to examine this perceptual construct. Research in psychophysics demonstrates a close interdependence between motion perception and perceived intentionality (Gao et al., 2009, 2010; Gao and Scholl, 2011; Gao et al., 2012; Parovel, 2023). Perceived animacy shapes people’s intentionality impressions in graphical stimuli, which are directly shaped by variations in motion principles such as direction and speed (Dittrich and Lea, 1994). Recent studies have shown that physical forces following the energy conservation hypothesis are the main cause of intentional perception (Vicovaro et al., 2023). The most compelling animated jumping-like motions tend to occur when the stimuli violate energy conservation and multiple bouncing cycles (Bingham et al., 1995; Parovel, 2023). Combined with recent observations, uncertainty is increasingly used in animated logos and becomes a key point affecting attention mechanisms. Inspired by this, we are interested in understanding whether motion principles with uncertain characteristics are the key factors in attracting sustained attention.

### 2.2 Perceived unpredictability and sustained attention

#### 2.2.1 Perceived unpredictability

“Most synonyms of the word uncertainty have decidedly unpleasant connotations. People gather facts, form opinions, and generate theories in an attempt to transform the unknown into the known—to make the world a bit less puzzling and more predictable by reducing their uncertainty about it” (Wilson et al., 2005). There is certainty that uncertainty is aversive most of the time, so people are forced to understand the causes of events and make them more predictable (Buhr and Dugas, 2002; Wilson et al., 2005). Indeed, much of the research in designs is driven by this objective. Whether brands are based on print or digital media, they all attempt to enhance the user experience through design. These studies are too numerous to enumerate here. Research has indicated that predictable events facilitate replication, but they may reduce novelty and engagement a phenomenon commonly referred to as the “pleasure paradox” (Wilson et al., 2005). Most novel and unexpected events fail to attract attention. People tend to focus on unexpected events that are relevant to their goals. Attention to such events usually elicits a relatively strong emotional response (Bernstein, 1969; Maltzman, 1979; Ben-Shakhar et al., 1989). Cognitive expectancies theory makes a similar point that disconfirmation of important expectancies leads to increased attention to and processing of inconsistent information (Olson et al., 1996). Inspired by these studies, we focus on the impact of uncertainty in animated logo design on user attention mechanisms.

#### 2.2.2 Perceived unpredictability of animated logos

Our previous research used eye-tracking to validate the influence of single-factor motion principles such as direction, speed, trajectory, and turning points on visual attention in animation design. The results showed that animation designs featuring vertical motion directions reflecting elasticity and gravity, variable motion speeds, curved motion trajectories, and a greater number of turning points attracted more user attention (Guan and Cho, 2024a, 2024b). These effective motion principles exhibit similar internal consistency, meaning that the diversity of changes prevents users from predicting the dynamic trends of the graphics. That forces users to constantly adjust their attention allocation strategies and devote more selective attention to alleviate this cognitive dissonance (Wilson et al., 2005; Polyportis et al., 2020). Therefore, we believe that using specific motion principles in animated logos can enhance users’ experience of uncertainty. This uncertainty stems from complex motion principles but differs from the definition of logo complexity. Complexity is evaluated from the perspective of visual representation, including the number of visual elements, irregularity, uniqueness, detail, and asymmetry (Van Grinsven and Das, 2016; Mahmood et al., 2019; Tang et al., 2025). The focus of this study is to evaluate from the perspective of motion principles such as speed, direction, and trajectory. A clear conceptual definition is beneficial to understanding the intrinsic mechanisms by which animated logos attract user attention. Therefore, we introduce physics concepts into the field of animation design, using “unpredictability” to represent the unknown, uncertain, and unexpected dynamic experiences that animated logos (Caves and Schack, 1997).

Through case analysis, we found that unpredictability is quite common in actual animated logos. IKEA’s animated logo frequently employs blue dot bouncing paths, font scaling changes, particle 360-degree circular paths, and background area variations. Yahoo’s animated logo repeatedly uses bouncing, scaling, circular paths, and panning movements at varying speeds. These fast-moving animations convey a lot of information in a short time, making it difficult for users to absorb it. The resulting cognitive bias increases the unpredictability of animated logos. Therefore, our study explored the unpredictability of animated logos to narrow the gap between theory and reality about perceived unpredictability in the field of animated logos.

#### 2.2.3 Clarification of the concept of perceived unpredictability

De Melo (2018) defines unpredictability as a critical component of enhancing design personalization through serendipitous experience. The author categorizes the sources of unpredictability in digital media design into algorithmic and artificial unpredictability, emphasizing events that exceed user expectations and anticipated goals. Scholars explored the positive impact of algorithmic unpredictability on fluid animations such as splashing waves, rolling smoke, and fluttering character hair in Hollywood films (Gowanlock, 2021), which is of great reference value for our study. However, algorithm-generated animations are unpredictable, and the two generated animations can differ significantly. Animated logos are pre-designed based on brand feature continuity and cyclicality rather than having absolute randomness like animations generated by algorithms. To distinguish these two concepts, this study defines artificial unpredictability as “perceived unpredictability” based on the characteristics of animated logos. Our goal is to observe the impact of animated logos on user experience by manipulating the level of perceived unpredictability.

#### 2.2.4 The influence of perceived unpredictability on sustained attention

The study of sustained attention was introduced by Mackworth (1948) and later developed into a scientific discipline by the human factors research community. Researchers believe that sustained attention is a limited resource that may be depleted (Scerbo et al., 1987), its time frame typically fluctuates from seconds to minutes (Esterman et al., 2013), and physiological arousal is crucial for sustained attention. Esterman and Rothlein (2019) cited a classic inverted U-shaped function to describe the relationship between performance and arousal (Yerkes and Dodson, 1908). Previous studies have shown that unexpected events increase people’s physiological arousal (Berlyne, 1960; Price and Geer, 1972; Le Poire and Burgoon, 1996; Wilson et al., 2005), that kind of arousal may intensify emotional responses to the event (Schachter and Singer, 1962; Zillmann, 2018). The study by de Melo (2018) pointed out that unpredictable elements can break monotonicity and activate the cognitive system of users. Cognitive dissonance theory also proposes the view that individuals need cognitive consistency. Therefore, individuals tend to take action to bridge the information discrepancy to reduce the inconsistency actively (Festinger, 1962). This theory provides evidence for the assumption that unpredictability attracts users’ attention. Meanwhile, resource-control theory and cognitive load theory emphasize the limited nature of sustained attention resources. When the cognitive demands of stimulus processing exceed the available mental load level, the maintenance of sustained attention will be impaired (Sweller, 1988; Thomson et al., 2015). Based on this, it is reasonable to speculate that there is an inverted U-shaped curve relationship between unpredictability and sustained attention. Therefore, we propose the following hypothesis:

*H1*. The relationship between perceived unpredictability and sustained attention shows an inverted U-shaped curve.

### 2.3 The mediating effect of perceived novelty

Novelty is the key determinant in marketing. Products regarded as novel tend to perform better in terms of sales and profits (Mukherjee and Hoyer, 2001; Gourville, 2006), and lead to stronger corporate performance (Mukherjee and Hoyer, 2001; Sorescu and Spanjol, 2008). The evidence provided by Sokolov (1963) indicates that people are more likely to pay attention to novel and unexpected events. Some studies have shown that attributes such as angles of ad images and incomplete fonts and logos enhance the novelty of a design (Folkes and Matta, 2004; Peracchio and Meyers-Levy, 2005; Hagtvedt, 2011). Kim and Lakshmanan (2015) found that the measurement of galvanic skin response that variable logos (such as Google holiday doodles) enhance users’ perceived novelty of the brand and trigger higher user emotional arousal. These insights reveal the intrinsic connection between perceived novelty and user attention in brand design. We have reason to expect that perceived novelty will have an impact on users’ sustained attention. However, the influence of novelty on sustained attention is not always positive. Walsh et al. (2011) proposed from the perspective of cognitive fluency theory that non-traditional logo designs significantly enhance users’ perceived novelty of the brand. However, such designs can break cognitive inertia and reduce the speed of brand recognition. Therefore, Walsh et al. suggest that attention should be paid to the balance between novelty and brand recognition efficiency when innovating brands. These insights reveal the intrinsic link between perceived novelty in brand design and user attention. We have reason to expect that perceived novelty has an impact on sustained attention.

Novelty and uncertainty are both powerful drivers of exploration (Hassall and Hunt, 2022; Cockburn et al., 2022). People often tend to be novelty seeking because new stimuli in the environment may yield greater rewards. In contrast, people’s initial attitudes toward uncertainty vary widely because uncertainty may lead to negative biases (Hassall and Hunt, 2022). Hassall and Cockburn’s research confirmed that resistance to negative signals of uncertainty is inhibited through optimistic novelty seeking. In other words, seeking novelty brings significant potential rewards, greatly reducing people’s perception of negative biases toward uncertainty. Another study on psychological control and novelty seeking directly demonstrates that thinking about an unpredictable world increased the perceived importance of preparedness for an unknown future and in turn the exploration of novelty seeking (Min and Schwarz, 2022). Based on these, we reasonably expect that users’ perceived unpredictability of animated logos will stimulate their behavior of novelty seeking and generate a perceived novelty, thereby encouraging users’ sustained attention to animated logos. Therefore, we propose the following hypothesis:

*H2*. Perceived novelty has a mediating effect on the impact of animated logos on users’ sustained attention.

*H2a*. The perceived unpredictability of animated logos affects users’ sustained attention through perceived novelty.

### 2.4 The association effect of brand types based on FCB grid

Attracting sustained attention is the first step to trigger user purchasing behavior, and users’ decision-making behavior shows significant differences according to product categories (Bandyopadhyay et al., 2009; Jeng, 2017). There is a very classic product categorization theory in marketing, the FCB grid (Vaughn, 1980). This grid contains two dimensions: involvement (High/Low) and information processing method (Think/Feel).

The information processing method (Think/Feel) is based on McGuire’s cognitive and affective classification, which originated from the utilitarian and value expressive functions in the functional attitude theory proposed by Katz (1960) and Choi et al. (2012). Katz believes that the persuasive effect of information is most powerful when the appeal information matches the underlying psychological motivation. This idea of matching brand types with psychological appeals is called the match-up hypothesis or functional matching (Lavine and Snyder, 1996; Shavitt and Nelson, 2002; Paek et al., 2010; Shavitt, 1989).

“Involvement” is a key factor in determining user behavior. High-involvement (abbreviated as Hi in the following text) are described as unusual, difficult to understand, or risky; while low-involvement (abbreviated as Li in the following text) are described as commonplace, easy to use, or involve minimal risk (Ratchford, 1987). Researchers (Laurent and Kapferer, 1985; Mittal and Lee, 1988; Mittal, 1989, 1995) further classified user participation behavior into product category involvement and purchase decision involvement. Product category involvement refers to an individual’s interest in a product, reflecting the user’s persistent willingness to pay attention to the product. Therefore, product category involvement is usually regarded as a long-term attachment. Purchase decision involvement refers to an individual’s interest in a purchase activity, reflecting the user’s situational attention to the purchase decision task, usually regarded as a short-term attachment (Bloch, 1981; Laurent and Kapferer, 1985; Michaelidou and Dibb, 2008). Animated logos are representative symbols of brand assets and reflect the long-term attachment between users and brands. Therefore, this study selects product category involvement as the grid dimension. Using more specific terms instead of generic ones will make the dimensions more explicit and easier to understand.

In the digital media era, the way brands and consumers interact has undergone significant changes. Product types have shifted from physical to virtual, and marketing strategies have transitioned from traditional offline to a combination of online and offline. To expand the market segments, brands no longer focus solely on utilitarian or hedonistic products but aim to build versatile brands. The utilitarian/hedonistic dichotomy is insufficient for precise brand segmentation. For example, Wuling MINI launched a family sedan in 2020 that supports “skin customization,” allowing users to regularly change the theme of their sedan like in a video game. This strategy quickly won the favor of young consumers, propelling MINI to consecutive quarters as the sales champion. Such actual examples of breaking traditional brand categorization and striving to create all-around brands are becoming increasingly common. Based on this opportunity, Cheong and Cheong (2021) restructured the FCB grid according to the characteristics of brands in the media age, shifting the focus from product categorization to brand categorization. According to this study, the first quadrant of the FCB grid (Hi/thinking) is described as Informative, the second quadrant (Hi/feeling) is described as Affective, the third quadrant (Li/thinking) is described as Habit Formation, and the fourth quadrant (Li/feeling) is described as Self-Satisfaction. Among them, Informative and Habit Formation still retain the characteristics of the think, while Affective and Self-Satisfaction retain the characteristics of the feel. Ratchford (1987) conceptualized the cognition-based think dimension as “utilitarian needs,” emphasizing functional attributes of products that are processed and evaluated through cognitive reasoning. The emotion-based feel dimension pertains to products related to self-satisfaction, social acceptance, and sensory stimulation, focusing on emotional appraisal. Informative and Affective both involve high involvement, meaning that people need to invest more cognitive resources in evaluating the brand. Habit Formation and Self-Satisfaction, on the other hand, mean that people make purchasing decisions without investing too much effort. This refined approach to brand categorization is crucial for the unpredictability of animated logos. Therefore, our research draws on Cheong’s brand categorization.

According to the match-up hypothesis, Kempf (1999) explained that value expression functions more closely related to emotional arousal, while utilitarian functions are more closely related to brand cognition. Utilitarian products are more rationally attractive, provide more cognitive-oriented benefits (Woods, 1960). While hedonic products are primarily consumed for expressive and emotional purposes, they are likely to be perceived as more complex than utilitarian products (Holbrook and Hirschman, 1982) and have higher internal consistency with unpredictable animated logos. When the predictable animated logo corresponds to the brand of a think product, its perception mechanism is cognitive, and the brand type is consistent. While the unpredictable animated logo corresponds to the brand of a felt product, the brand type is consistent. Therefore, we predict that unpredictable animated logos are more effective for feel brands with affective characteristics. Because the psychological experience of surprise and change brought about by unpredictable animated logos aligns with the feel brands. In contrast, predictable animated logos are clearer in terms of logic and closely related to the think brand. Additionally, the consistency between involvement and unpredictability also influences the effectiveness of animated logos. For example, gems, perfumes, tobacco, and games are typically perceived as feel, while cars, credit cards, batteries, and pesticides are perceived as think (Cheong and Cheong, 2021). However, compared to high-involvement brands like gems and cars, using unpredictable animated logos in brands like tobacco, games, batteries, and pesticides might be more effective, because engaging with high-involvement brands typically requires significant cognitive resources for information processing (Cheong and Cheong, 2021). Unpredictable animated logos require users to devote resources, which may lead to cognitive load. Therefore, we predict that using unpredictable animated logos is more effective for brands with low-involvement than for those with high-involvement. Therefore, we propose the following hypothesis:

*H3*. Brand types have a moderating effect on the impact of animated logos on users’ sustained attention.

*H3a*. For think brands such as Informative or Habit Formation, the perceived unpredictability of animated logos has a negative moderating effect on users’ sustained attention.

*H3b*. For feel brands such as Affective or Self-Satisfaction, the perceived unpredictability of animated logos has a positive moderating effect on users’ sustained attention.

*H3c*. For high-involvement brands such as Informative and Affective, the perceived unpredictability of animated logo has a negative moderating effect on users’ sustained attention.

*H3d*. For low-involvement brands such as Habit Formation and Self-Satisfaction, the perceived unpredictability of animated logos has a positive moderating effect on users’ sustained attention.

## 3 Study 1

The main objective of this study is to investigate the impact of animated logos’ perceived unpredictability on users’ sustained attention while controlling for other animated logo characteristics to provide evidence in support of H1. The second objective is to explore whether perceived unpredictability in animated logos affects users’ sustained attention through perceived novelty and provide evidence supporting H2. The third objective is to examine whether the impact of perceived unpredictability on perceived novelty and users’ sustained attention varies by brand type to provide evidence supporting H3.

### 3.1 Materials

We randomly selected 100 animated logo clips from public websites and verified the availability through the global brand database (WIPO). Product categories covered include media and entertainment, lifestyle services, e-commerce, business management, retail manufacturing, food and beverage, sports, apparel, design, finance, legal services, intelligent information technology, and other industries. Well-known brands were excluded from our sample to avoid potential bias in user attitudes and behaviors caused by the influence of these highly established brand assets. In addition, existing research has determined three categories of brand logos: pure graphics, pure text, and a combination of graphics and text (Bresciani and Del Ponte, 2017; Peng et al., 2023). According to the experimental materials, the combination of graphics and text in animated logos was the most common form. Therefore, we eliminated 14 clips of pure graphic animation logos and 23 clips of pure text animation logos. The remaining 63 animation logos contain graphics and text as experimental materials. Prior research on static logo design has identified design features that influence user attention, including logo color (Lin et al., 2016; Liu et al., 2021; Shen et al., 2021; Huang et al., 2023; Yang et al., 2024), background color and background area (Lewandowska and Olejnik-Krugly, 2021; Shao et al., 2024), and logo saturation (Yu and Ouyang, 2024; Deng et al., 2022). Therefore, we used Adobe After Effects to uniformly adjust the overall size, color, saturation, and background color of animated logos. Use the invert function to convert animated logos with a dark background to a light background. The inherent characteristics of the logo were discarded, and only the animation presentation has been retained by the principle of fair use. Participants were like watching these animated logos through black-and-white television. See Supplementary material for examples of these clips.

### 3.2 Experimental procedure

This study recruited a total of 128 participants for a repeated measures in Jinan, China, and Jeonju, South Korea (Male = 56, Female = 72; M age = 29.836 years, SD = 5.655, Age range = 19–47 years). A total of 1,920 questionnaires were collected. After eliminating 76 incomplete and irregular questionnaires, 1,844 valid questionnaires remained. The study has been approved by the relevant ethics committee (2025-03-017-001). Before the investigation began, the researchers asked the subjects for basic information and informed them of the experimental procedures. After confirming that the participants understood and agreed to the experimental content, they were guided to watch the animated videos and complete the corresponding questionnaires (conducted separately for the control and experimental groups). Due to the large number of experimental materials, we informed participants that they could randomly select 15 materials for evaluation to avoid fatigue. The experimental materials that first reach the planned sample size will be suspended from display to ensure the average coverage of the sample size.

**Figure 1:**
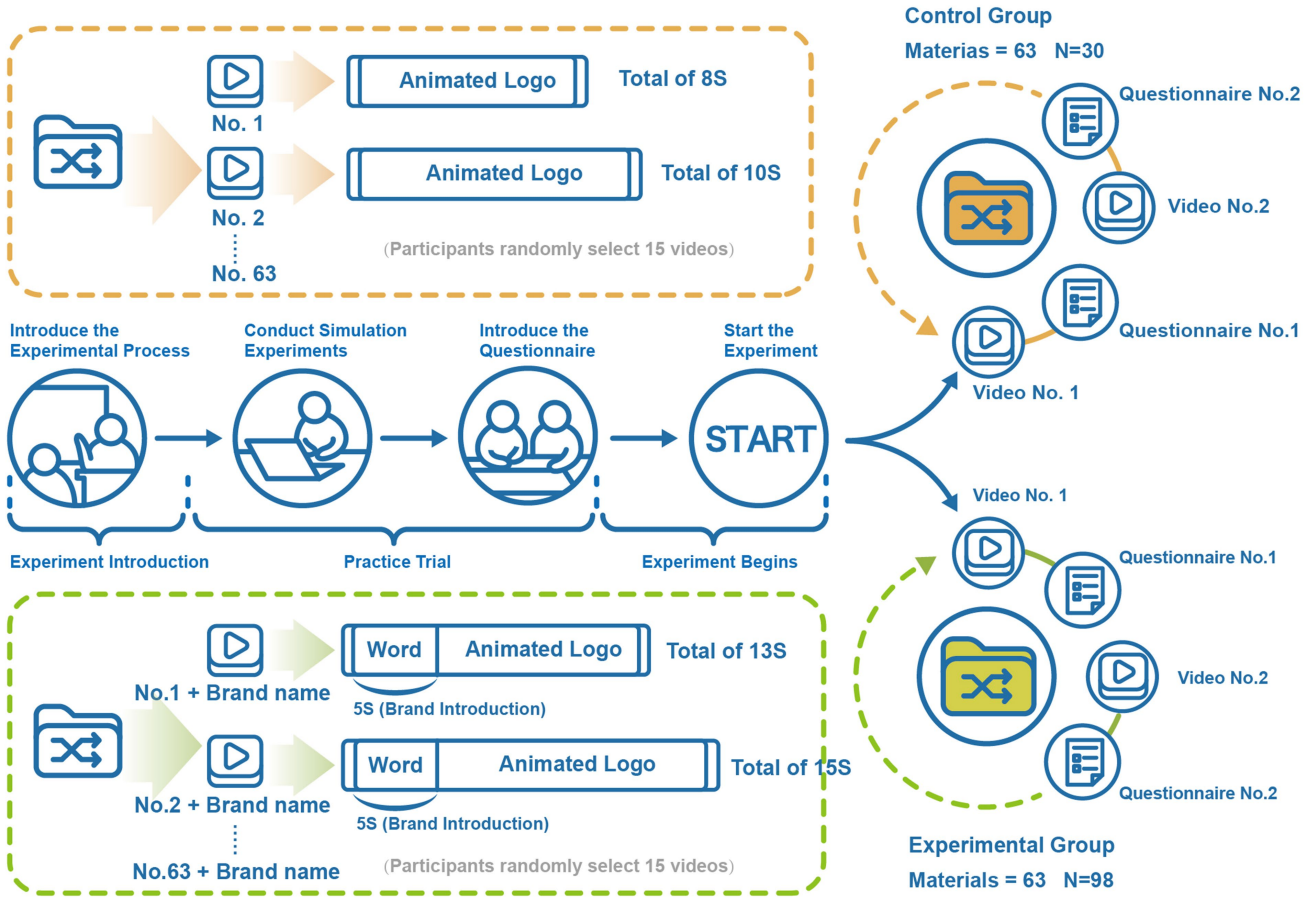
Schematic representation of study 1.

We observed the moderating effect of brand types through a between-subjects controlled experiment. As shown in Figure 1. We prepared two groups of animated logo videos as stimulus materials. The control group contained 63 animated logo videos. Since the materials were derived from actual cases, the duration of each video ranged from 6 to 11 s and did not contain any additional prompts about brand categorization. The experimental group added a 5-s opening text description to the stimulus material of the control group, and the material was named “number + brand name” to ensure that the subjects had sufficient time to understand. After watching the video, participants need to complete a questionnaire with the same number accordingly. The questionnaire includes the participants’ assessment of perceived unpredictability, perceived novelty, and sustained attention to animated logos. The questionnaire for the experimental group also included six items related to brand type evaluation, which served as the basis for our coding of brand types.

According to the questionnaire results, we classified 63 brands into Informative (Hi/Think), Affective (Hi/Feel), Habit Formation (Li/Think), and Self-Satisfaction (Li/Feel). To ensure that there were sufficient samples for each level, we distributed questionnaires to the participants in the control group and the experimental group at a ratio of 1:3.5.

### 3.3 Variable selection

The independent variable is the user’s assessment of the perceived unpredictability of animated logos. Integrating several studies of complex perception (Forsythe et al., 2008; Palumbo et al., 2014; Kyle-Davidson et al., 2023) and Alter and Oppenheimer’s (2009) theory of cognitive fluency, four items on perceived unpredictability are proposed. Referring to a cross-cultural by Jeong and Park (1997), the novelty scale is integrated into two items to assess the perceived novelty of animated logos. The dependent variable is the user’s sustained attention to animated logos, which is evaluated by four items. These items are based on the self-report attention scale of Chen et al. (2023) and the brand-related social media research of Eroglu et al. (2003). The six items of the moderating variables refer to the FCB grid scale summarized by Cheong and Cheong (2021) based on the characteristics of the digital age. The questionnaire items are shown in Table 1. All items are measured using a Likert seven-point scale.

**Table 1:** Scale items: brand type, perceived unpredictability, perceived novelty, and sustained attention.

Variable	Item	Scale anchors	Cronbach’s alpha
Brand type (Categorical)	If I decide to choose this brand, this decision for me is:	Very unimportant—Very important	0.96
	Before choosing this brand, my thoughts are:	Little of thought—Lot of thought	
	If the brand makes a mistake, the loss to me is:	Little to lose—Lot to lose	
	When I use this brand:	Not on functionality—Based on functionality	0.873
	When I use this brand, its value comes primarily from the senses (looks, taste, touch, smell, or sounds):	Strongly disagree—Strongly agree	
	Using this brand helps me express my personality:	Not on personality—Based on personality	
Perceived unpredictability	The motion direction of the graphic is unpredictable:	Strongly disagree—Strongly agree	0.889
	The motion speed of the graphic often changes:	Strongly disagree—Strongly agree	
	The motion trajectory of the graphic is irregular:	Strongly disagree—Strongly agree	
	The animation presentation surprised me:	Strongly disagree—Strongly agree	
Perceived novelty	The animation presentation is novel to me:	Strongly disagree—Strongly agree	0.906
	I have never seen such animation presentation before:	Strongly disagree—Strongly agree	
Sustained attention	I am willing to spend more time watching this animated logo:	Strongly disagree—Strongly agree	0.919
	I noticed some subtle changes in this animated logo:	Strongly disagree—Strongly agree	
	I would like to see this animated logo again:	Strongly disagree—Strongly agree	
	I am rarely distracted by other things while watching:	Strongly disagree—Strongly agree	

Our stimulus materials are taken from actual cases, so there are many differences among each animated logo. We have uniformly adjusted the color, brightness, scale, and layout of the animated logos during the preparation stage. However, there are still some confusing factors that cannot be achieved through uniform adjustment. We invited three brand experts to code these confounding factors as control variables. All three experts hold master’s degrees or higher in design and are unaware of our current study. The coding standard of the control variable based on the research of the existing literature (McDougall et al., 1999; Collaud et al., 2022; Mu et al., 2022; Eyni et al., 2023; Shao et al., 2024; Zhang et al., 2024), including concreteness (1 = Low, 3 = High), complexity (1 = Low, 3 = High), semantic relevance (1 = Low, 3 = High), and visual thickness (1 = Thin, 3 = Thick). These potential impacts are translated into specific indicators by coding control variables.

### 3.4 Results

#### 3.4.1 Robustness checks

Table 2 presents the results of model robustness checks output using SPSS. The residual distributions of the key variables approximate normality, indicating that the data meet the requirements of mediating regression analysis. The results of the Pearson correlation test were also as expected, with each variable showing a significant positive correlation with one another. Given the high correlations among key variables, we conducted collinearity diagnostics after including control variables. The results indicated no multicollinearity (average VIF = 1.390; all VIFs ≤ 2.126), supporting the subsequent analyses.

**Table 2:** Robustness check: means, standard deviations, and correlations of the main study variables.

Variable	M	SD	Skewness	Kurtosis	1	2	3	4
1. Perceived unpredictability	4.409	1.448	−0.269	−0.747	1.000			
2. Perceived novelty	4.514	1.475	−0.303	−0.640	0.703**	1.000		
3. Sustained attention	4.550	1.393	−0.241	−0.793	0.600**	0.806**	1.000	
4. Brand type (Categorical)	2.073	1.467	−0.233	−1.342	0.083**	0.126**	0.112**	1.000

*N* = 1,844. Independent variable = perceived unpredictability; Mediating variable = perceived novelty; Dependent variable = sustained attention; Moderating variable = brand type. **p < 0.01; *p < 0.05.

#### 3.4.2 Mediation regression analysis

**Figure 2:**
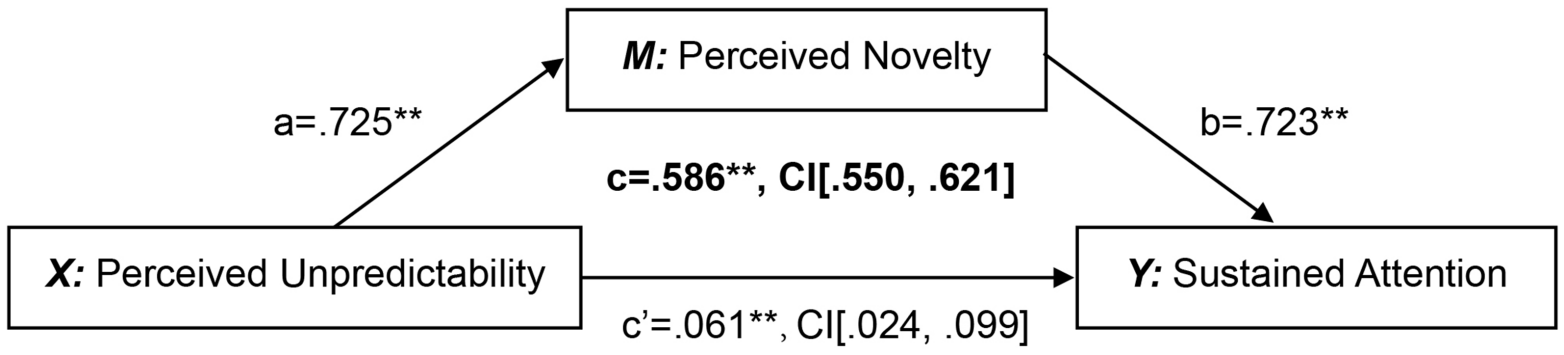
The statistical diagram of the mediation model of perceived novelty.

We performed mediating regression analysis with concreteness, complexity, semantic relevance, and visual thickness as control variables. The results are presented in Table 3. Although Model 1 explanatory power has been limited, the inclusion of control variables has helped mitigate the bias caused by brand differences. Therefore, these variables were retained in the subsequent analysis. After incorporating perceptual unpredictability, the explanatory power of Model 2 significantly improved. Perceptual unpredictability had a significant positive impact on sustained attention, indicating that perceptual unpredictability independently predicts changes in users’ sustained attention to animated logos. After incorporating perceived novelty into Model 4, the explanatory power was further enhanced. At this point, the perceived novelty had a significant positive impact on users’ sustained attention. The influence of perceived unpredictability on users’ sustained attention remains substantial, but the effect force weakens. Subsequently, we verified the significant impact of unpredictability on perceived novelty using the SPSS PROCESS macro (Model 4) by Hayes (2017). The overall results indicate a substantial direct effect of perceived unpredictability on sustained attention. However, the direct effect of perceived unpredictability was substantially reduced after incorporating the perceived novelty, suggesting a potential mediating effect (please see Figure 2).

**Table 3:** Results of the mediation regression analysis.

Variable	Model 1: sustained attention (Y)		Model 2: sustained attention (Y)		Model 3: perceived novelty (M)		Model 4: sustained attention (Y)	
	B	SE	B	SE	B	SE	B	SE
Concreteness (C)	−0.049	0.043	0.102**	0.034	0.124**	0.032	0.013	0.025
Complexity (C)	0.000	0.041	0.008	0.033	0.098**	0.031	−0.063*	0.024
Semantic relevance (C)	0.122**	0.043	0.016	0.035	0.099**	0.032	−0.056*	0.026
Visual thickness (C)	−0.047	0.039	0.028	0.031	0.020	0.029	0.013	0.023
Perceived unpredictability (X)			0.586**	0.018	0.725**	0.017	0.061**	0.019
Perceived novelty (M)							0.723**	0.018
*F*	2.279		210.876**		378.383**		577.603**	
*R* ^2^	0.005		0.365		0.507		0.654	

*N* = 1,844. X = Independent variable; Y=Dependent variable; M = Mediation variable; C=Control variables.

C is categorical variable, which is coded into three levels. X, M, and Y are continuous variables, and they are all centered. **p < 0.01; *p < 0.05.

To further verify the robustness of the mediation effect, we ran a Bootstrap analysis based on 5,000 repeated samplings (please see Table 4). Effect size calculations revealed that the indirect effect was 0.524, with a 95% confidence interval that excluded zero [0.487, 0.562], indicating statistical significance. The direct effect was 0.061, and its 95% confidence interval also excluded zero [0.024, 0.099], further supporting the result of a partial mediation effect.

**Table 4:** Effect proportion: total effect, direct effect and indirect effect.

Effect type	Effect	BootSE	BootLLCI	BootULCI	Effect proportion
Total effect	0.586	0.018	0.550	0.621	
Direct effect	0.061	0.019	0.024	0.099	10.41%
Indirect effect	0.524	0.019	0.487	0.562	89.42%

In spite of the study demonstrates the mediating effect of perceived novelty and provides a new perspective on the attraction mechanism of animated logos to users’ sustained attention. However, we still need to remain vigilant about this result. Since our experimental materials were taken from actual cases, the measurement results may be greatly affected by brand types. Studies have proved that brand type consistency affects users’ brand attitudes during the marketing process (Johan Lanseng and Erling Olsen, 2012; Šerić et al., 2020). We recognize that differences in brand type were an important factor in perceived novelty and sustained attention. Therefore, we incorporated brand types as a moderating variable and performed a moderated mediation regression analysis to determine whether the brand type had a moderating effect.

#### 3.4.3 Moderated mediation regression analysis

We used Model 59 of the SPSS PROCESS macro by Hayes (2017) to verify the moderating effect of brand type. According to the experimental design, brand type was used as a 5-level multi-category moderator variable, including a control group with no intervention and four experimental groups with Informative, Affective, Habit Formation, and Self-Satisfaction as intervention conditions. We used the control group as the baseline for virtual coding of the moderating variables. The main and interaction effects coefficients of Informative, Affective, Habit Formation, and Self-Satisfaction were estimated based on the control group. To verify the appropriateness of the control group as a baseline, we performed a one-way ANOVA. Table 5 shows that there were significant differences between the control and the experimental group in terms of the effects of sustained attention. *Post hoc* comparisons revealed that Habit Formation and Self-Satisfaction were significantly lower than the control group, Affective was significantly higher than the control group, and Informative showed no significant difference from the control group. Considering the overall significance of the differences and the inherent theoretical of the control group, we retained the control group and used it as the baseline for subsequent analyses.

**Table 5:** One-way ANOVA and post hoc comparisons: effectiveness of brand type grouping.

Brand type grouping	Mean	SD	One-way ANOVA	*Post Hoc* test
Control Group	4.5566	1.50652	25.143**	Control Group < Affective Control Group > Habit Formation Control Group > Self-Satisfaction
Informative	4.4441	1.38156		
Affective	5.1458	1.29585		
Habit Formation	4.2366	1.08724		
Self-Satisfaction	4.2281	1.29185		

*N* = 1,844. **p < 0.01; *p < 0.05.

**Figure 3:**
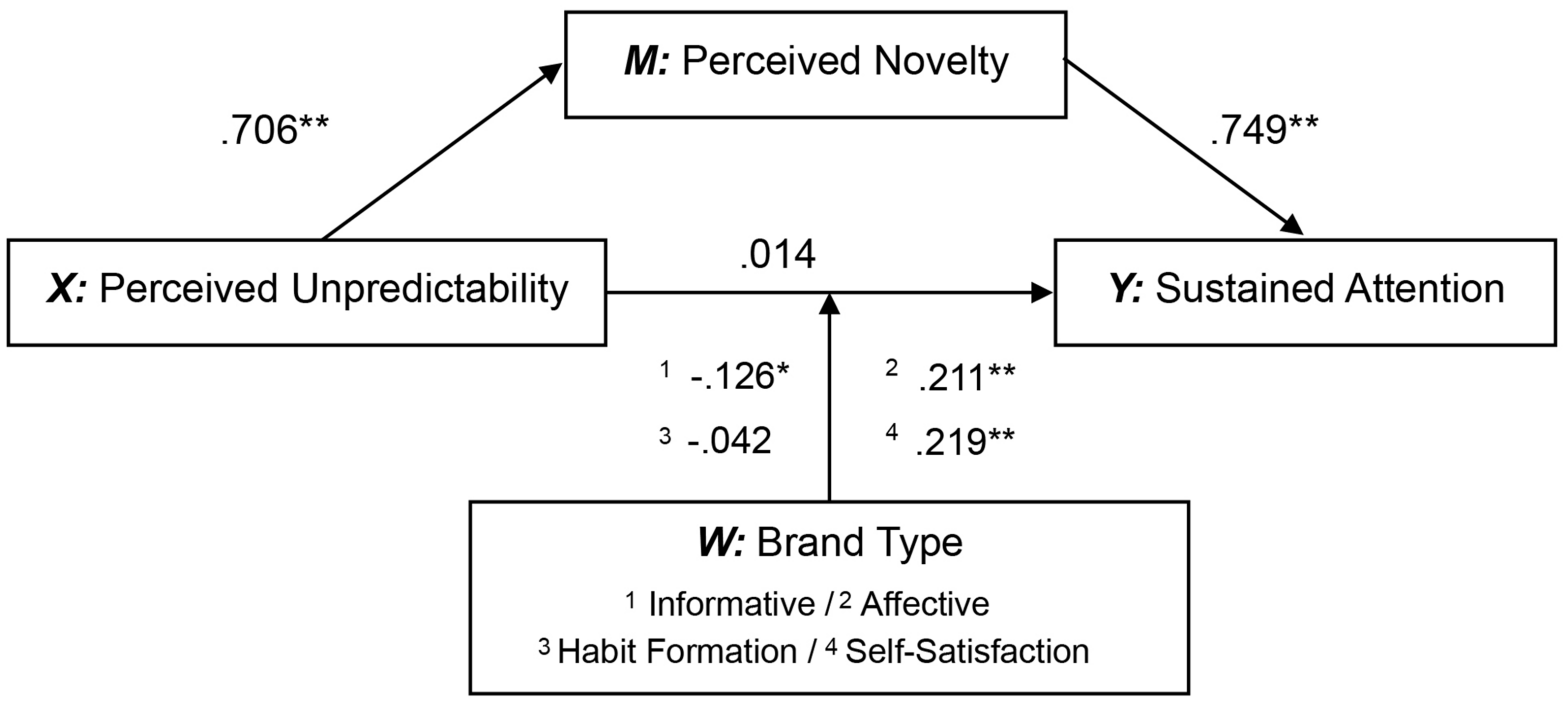
The statistical diagram of the moderated mediation model of brand type.

The results shown in Table 6 indicate that the mediation pathway was significantly moderated by brand types, suggesting that the strength of the mediating effect varies across different brand types. Under the Affective and Self-Satisfaction, brand types significantly and positively moderated the relationship between unpredictability and sustained attention. Under the Informative, brand types showed a significant negative moderating effect. In contrast, the moderating effect of brand types was not significant in habit formation, although the relationship between unpredictability and sustained attention remained negative (please see Figure 3). Analyzing the impact of perceived novelty on sustained attention also revealed a moderating effect of brand types. Under the Affective and Self-Satisfaction, brand types significantly and negatively moderated the relationship between perceived novelty and sustained attention. While under the Informative variable, the result was just the opposite.

**Table 6:** Results of the moderated mediation regression analysis: brand type based on FCB grid.

Variable	Perceived novelty (M)		Sustained attention (Y)	
	B	SE	B	SE
Concreteness (C)	0.088**	0.032	0.014	0.025
Complexity (C)	0.095**	0.030	−0.059*	0.024
Semantic relevance (C)	0.088**	0.032	−0.056*	0.026
Visual thickness (C)	0.020	0.028	0.008	0.023
Perceived unpredictability (X)	0.706**	0.029	0.014	0.032
Habit formation (low-involvement/think) (W)	0.034	0.096	−0.148	0.076
Self-satisfaction (low-involvement/feel) (W)	−0.003	0.074	−0.196**	0.059
Informative (high-involvement/think) (W)	−0.118	0.065	−0.051	0.052
Affective (high-involvement/feel) (W)	0.495**	0.073	0.092	0.061
Perceived unpredictability × habit formation	−0.033	0.074	−0.042	0.076
Perceived unpredictability × self-satisfaction	0.01	0.05	0.219**	0.057
Perceived unpredictability × informative	0.104*	0.043	−0.126*	0.05
Perceived unpredictability × affective	−0.126*	0.049	0.211**	0.051
Perceived novelty (M)			0.749**	0.032
Perceived novelty × habit formation			−0.026	0.072
Perceived novelty × self-satisfaction			−0.197**	0.058
Perceived novelty × informative			0.101*	0.049
Perceived novelty × affective			−0.134*	0.054
*R* ^2^	0.531		0.668	
*F*	159.19**		204.301**	

*N* = 1,844. X = Independent variable; Y=Dependent variable; M = Mediation variable; W = Moderation variable; C=Control variables. C and W is categorical variable, which is coded into three levels. X, M, and Y are continuous variables, and they are all centered. **p < 0.01; *p < 0.05.

**Figure 4:**
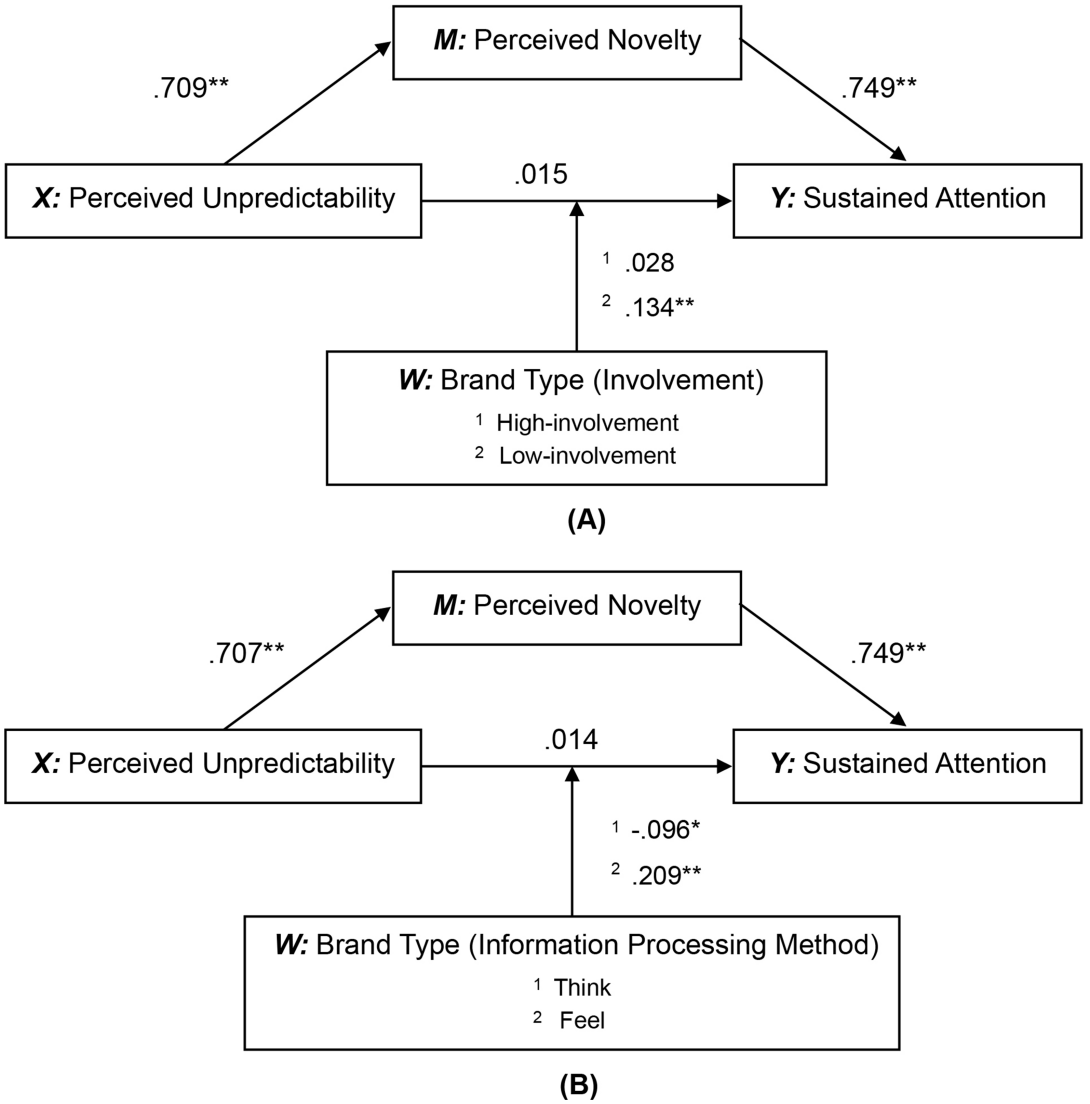
The statistical diagram of the moderated mediation model based on **(A)** involvement and **(B)** information processing method.

Specifically, Affective and Self-Satisfaction amplify the impact of unpredictability on sustained attention, while attenuating the impact of novelty on sustained attention. In contrast, Informative weakens the impact of unpredictability on sustained attention, but strengthens the impact of novelty on sustained attention. Particular attention should be paid to feel brands showed more significant moderating effects among the brand types classified based on information processing. To further clarify this result, we performed a moderated mediating regression analysis again, taking involvement (High/Low) and information processing method (Think/Feel) as moderating variables, respectively, (please see Figure 4). The results are shown in Table 7.

**Table 7:** Results of the moderated mediation regression analysis: involvement and information processing method.

Variable	Involvement				Information processing method			
	Perceived novelty (M)		Sustained attention (Y)		Perceived novelty (M)		Sustained attention (Y)	
	B	SE	B	SE	B	SE	B	SE
Concreteness (C)	0.125**	0.032	0.020	0.025	0.092**	0.032	0.011	0.026
Complexity (C)	0.102**	0.031	−0.058*	0.024	0.092**	0.031	−0.065**	0.024
Semantic relevance (C)	0.094**	0.032	−0.064*	0.026	0.094**	0.032	−0.049	0.026
Visual thickness (C)	0.021	0.029	0.009	0.023	0.023	0.029	0.009	0.023
Perceived unpredictability (X)	0.709**	0.030	0.015	0.033	0.707**	0.030	0.014	0.032
Perceived novelty (M)			0.749**	0.033			0.749**	0.032
Low-involvement (W)	0.006	0.068	−0.175**	0.053				
High-involvement (W)	0.120*	0.060	−0.021	0.047				
Perceived unpredictability × low-involvement	−0.004	0.046	0.134**	0.051				
Perceived unpredictability × high-involvement	0.027	0.039	0.028	0.043				
Perceived novelty × low-involvement			−0.133**	0.051				
Perceived novelty × high-involvement			0.003	0.042				
Think (W)					−0.067	0.062	−0.079	0.049
Feel (W)					0.232**	0.062	−0.043	0.049
Perceived unpredictability × think					0.070	0.041	−0.096*	0.046
Perceived unpredictability × feel					−0.025	0.041	0.209**	0.044
Perceived novelty × think							0.068	0.045
Perceived novelty × feel							−0.126**	0.045
*R*-sq	0.509		0.658		0.517		0.663	
*F*	211.23**		293.943**		217.938**		300.57**	

*N* = 1,844. X = Independent variable; Y=Dependent variable; M = Mediation variable; W = Moderation variable; C=Control variables. C and W is categorical variable, which is coded into three levels. X, M, and Y are continuous variables, and they are all centered. **p < 0.01; *p < 0.05.

A comparison of the moderating effects of involvement revealed that low-involvement brands enhance the impact of unpredictability on sustained attention while weakening the impact of novelty on sustained attention, which was consistent with the results we verified previously. Comparing the moderating effects of information processing methods revealed that feel brands enhanced the effect of unpredictability on sustained attention while weakening the effect of novelty, whereas the think brand only weakened the effect of unpredictability on sustained attention. That was also consistent with the results we verified previously.

### 3.5 Discussion

Although there was no inverted U-shaped relationship as expected, our results confirmed a positive linear relationship between perceived unpredictability and sustained attention. Therefore, H1 was partially supported. We realize that although actual animated logos more accurately reflect market performance, the unpredictability of these animated logos was not usually overly high. That resulted in our observed levels of perceived unpredictability falling short of the threshold for an inverted U-shaped curve. We have reasons to believe that when the perceived unpredictability increases, users’ sustained attention to animated logos may decrease. Such errors may lead us to recommendations that diverge substantially from empirical realities. Therefore, we further explored this issue in Study 2.

Mediating analysis verified the positive influence relationship between perceived unpredictability, perceived novelty, and users’ sustained attention. H2 and H2a were supported. As expected, perceived unpredictability affects sustained attention, which was consistent with the findings of Vicovaro et al. (2023). Unlike prior research, our findings revealed that the impact of perceived unpredictability on sustained attention was mediated by other underlying variables. After incorporating the mediating variable of perceived novelty, perceived novelty fully explains the direct impact of perceived unpredictability. Suggests that perceived novelty was an important feature that aroused users’ attention. This result also confirms the crucial role of perceived novelty in building a good marketing response (Kunz et al., 2011; Shams et al., 2015). In this phase of the study, we employed real marketing communication cases as experimental materials, carefully controlled for other features unrelated to perceived unpredictability, to ensure the validity of the results. This method was superior to examining one feature while ignoring the other (Zoghaib et al., 2023), making the data closer to reality, enhancing the credibility of the study results, and providing strong supporting evidence for existing related results and subsequent research.

The results of the moderated mediation analysis provided support for H3. The influence of perceived unpredictability and perceived novelty on sustained attention were both moderated by brand types. Put differently, when animated logos correspond to brand types with hedonistic tendencies such as Affective and Self-Satisfaction, the positive effect of unpredictability on sustained attention becomes stronger, while the positive effect of novelty becomes weaker. Conversely, when animated logos are associated with utilitarian brands such as the Informative, the impact of perceived unpredictability will weaken, while perceived novelty will increase. H3a and H3b were supported.

## 4 Study 2

To verify the robustness of Study 1, we set stricter experimental conditions in Study 2. In this study, we manipulated (rather than measured) the perceived unpredictability of animated logos to observe the changes in the results at higher levels of perceived unpredictability through eye tracking. Eye tracking has been widely applied in visual attention related to advertising appeal (Pieters et al., 2010; Myers et al., 2020; Hana Frade et al., 2025), brand memory (Simmonds et al., 2020), visual marketing strategies (Wedel and Pieters, 2006), and attention dynamics (Carter and Luke, 2020).

### 4.1 Materials

We ranked the stimulus materials in Study 1 based on questionnaire assessments of perceived unpredictability and selected five videos with high and low scores to analyze the motion principles. After disassembling the video frame-by-frame by Adobe After Effect, we found that the animated logos with high and low perceived unpredictability showed some specific patterns in motion principles. We describe the animation case and the analysis process in Supplementary material.

Overall, stimulus materials with higher perceived unpredictability exhibit faster speeds, greater amplitudes, more directions, and more complex structures. Based on these motion principles, we used Adobe After Effects to create three 10-s animated logos for a virtual brand and took the animation presentations as the sole manipulation variable. (see Supplementary material).

Subsequently, we invited three experts from Study 1 to independently validate the animated logo to examine whether there were significant differences in perceived unpredictability in the materials. Ensured that we observed the impact of perceived unpredictability on sustained attention under strict conditions. That is important because manipulating the independent variables while controlling the rest of the environment is needed to establish causality (Viglia et al., 2021).

### 4.2 Experimental procedure

We recruited 52 participants from Jeonju, South Korea, and Jinan, China (Male = 28, Female = 24; M age = 30.808 years, SD = 5.770, Age range = 23–45 years). Before the experiment, all participants read and signed an informed consent form and familiarized themselves with the experimental procedure under the guidance of the researchers. The formal experiments comprised eye-tracking tests and questionnaires.

**Figure 5:**
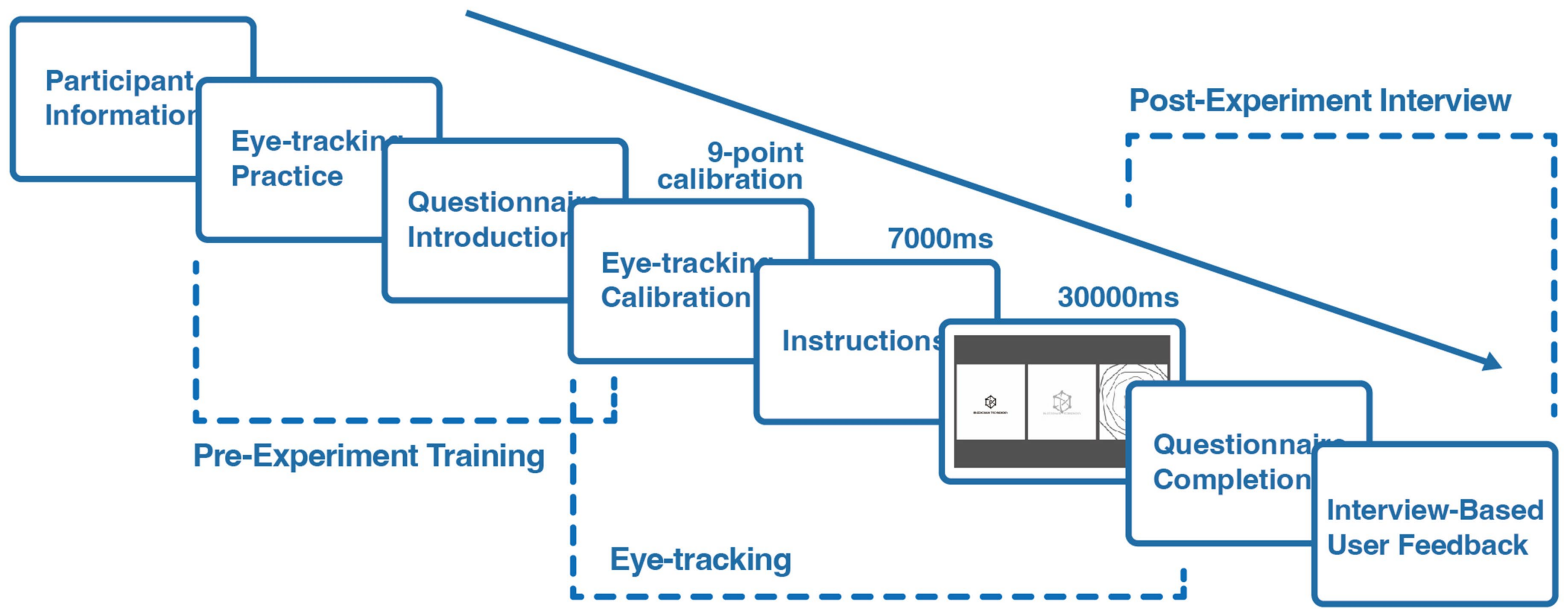
Experimental flowchart of study 2.

We conducted a within-subject repeated measurement experiment using a Tobii Pro Nano eye-tracker with a sampling rate of 60 Hz. The experimental program was written through Tobii Pro Lab to present the three animated logos simultaneously on the screen. Participants can independently complete the experiment based on text prompts, avoiding data confusion caused by human intervention during the experiment. After the participants confirm to start the experiment, three animation logos play simultaneously. Each animation clip was replayed three times, with a total duration of 30 s, ensuring that participants had sufficient time to freely view the screen. After the eye-tracking test, participants fill out relevant questionnaires to verify the credibility of the materials. The questionnaire used the same items as Study 1, including evaluations of perceived unpredictability, perceived novelty, and sustained attention toward animated logos. All items were measured using a seven-point Likert scale. The experimental process is shown in Figure 5.

### 4.3 Results

The indicators of eye-tracking are the total duration of fixation, number of fixation, number of return visits, and time to first fixation. Table 8 presents the definitions of eye-tracking indicators. The Shapiro–Wilk test results indicated that the residuals of the total duration of fixation, number of fixation, and number of return visits all passed the normality test (*p* > 0.05). Therefore, SPSS was used to perform a one-way repeated measures ANOVA of variance on the data.

**Table 8:** Definition of eye-tracking test indicators.

Indicators	Definition	Reflective behavior	Psychological significance
Total duration of fixation in AOI	The total time has fixated each AOl on all Media.	The intensity of information processing on AOI.	Willingness to sustain attention.
Number of fixation in AOI	The number of fixations within each AOl on all Media (excluding scan).	The number of information is processed on AOI.	Frequency of information processing.
Number of return visit in AOI	The number of fixations and scan after subtracting the first view within each AOL on all media.	The number of the AOI is returned for exploration.	Difficulty of information processing.
Time to first fixation in AOI	The time to first fixation for each AOl on all Media.	The time sequence when the AOI is first noticed.	Initial appeal of the material.

**Figure 6:**
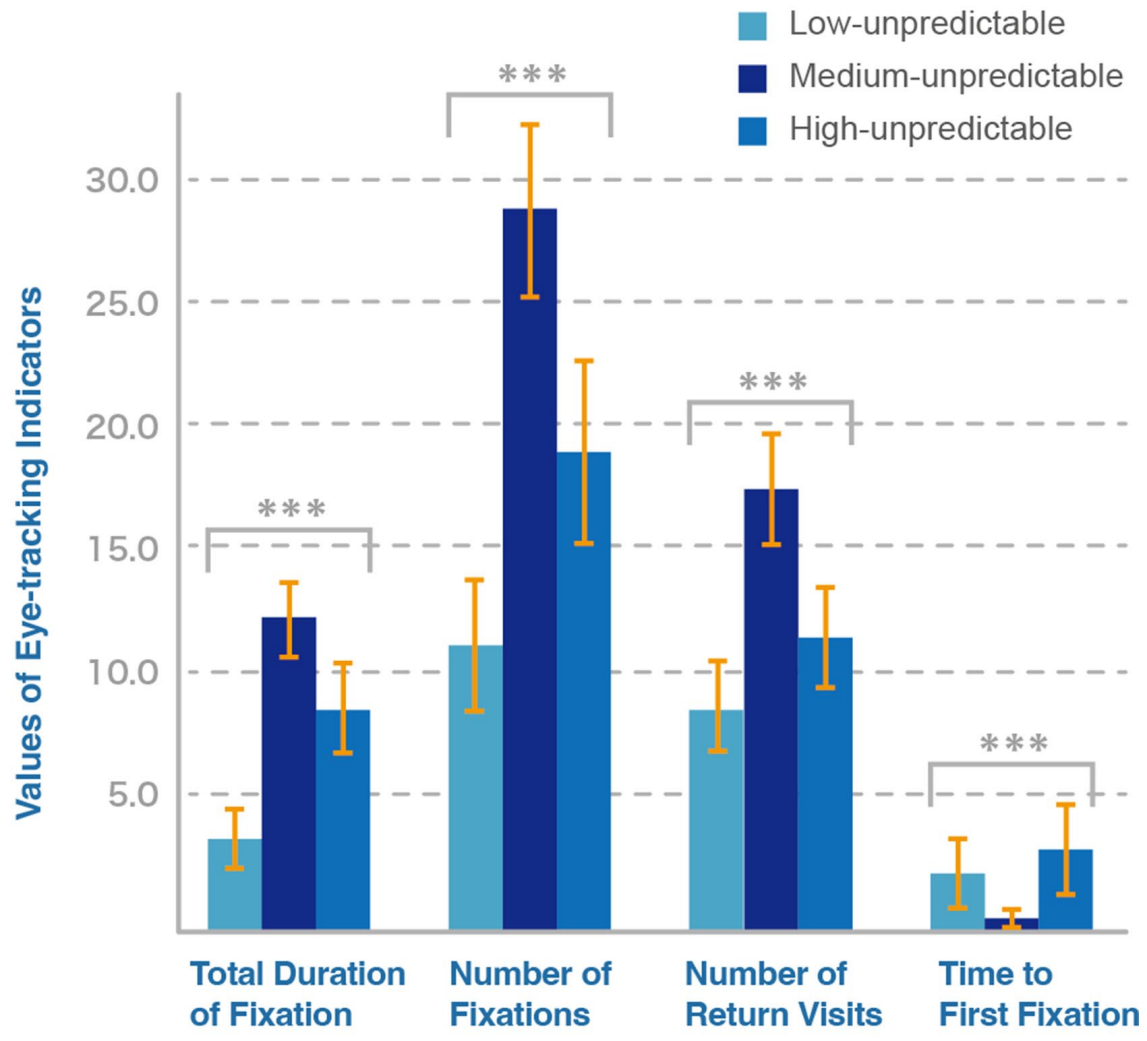
Comparison of eye-tracking result under each indicator.

The results indicated that perceived unpredictability showed significant differences in the three indicators of the total duration of fixation, number of fixation, and number of return visits (see Table 9). Among the total duration of fixation, the medium unpredictability condition (M = 11.564, SD = 2.803) resulted in a longer total fixation duration than the high unpredictability condition (M = 9.570, SD = 3.337) and the low unpredictability condition (M = 4.470, SD = 2.067), indicating that people have a stronger willingness to pay attention to animated logos with a medium level of unpredictability. The results for the number of fixation and return visits showed the same trend as the total duration of fixation. In other words, the animated logos with medium unpredictability levels performed best, followed by high unpredictability and low unpredictability. This indicates that people have the highest frequency of cognitive processing and the strongest degree of visual interest in animated logos with a medium level of unpredictability. Figure 6 reflects this trend more intuitively.

**Table 9:** Results of the eye-tracking test.

Eye-tracking indicators	Shapiro–Wilk test of residuals		Descriptive Statistics		Repeated measures ANOVA			*Post Hoc* test (Bonferroni)
	Perceived Unpredictability	sig.	Mean	SD	*F*	df	*η* ^2^	
Total duration of fixation	Low	0.847	4.470	2.067	63.151**	0.847	0.553	Medium > High > Low A = −14.075**, B = −7.834**, C = −2.586*
	Medium	0.128	11.564	2.803				
	High	0.578	9.570	3.337				
Number of fixations	Low	0.117	12.712	6.892	72.150**	0.941	0.586	Medium > High > Low A = −13.872**, B = −4.937**, C = −6.527**
	Medium	0.495	29.539	8.228				
	High	0.51	20.577	8.318				
Number of return visits	Low	0.627	7.865	3.684	125.656**	0.977	0.711	Medium > High > Low A = −16.020**, B = −5.858**, C = −10.355**
	Medium	0.387	16.789	5.965				
	High	0.521	11.539	4.474				

*N* = 52. A = Low/Medium; B = Low/High; C = Medium/High. Repeated measures ANOVA test results were corrected by Sinfert-Friedel, and post hoc test results were corrected by Bonferroni. **p < 0.01; *p < 0.05.

The *post hoc* test corrected using the Bonferroni method revealed that in the low unpredictability condition, all indicators were significantly lower, which was consistent with the results of H1 in Study 1. Regarding the indicator of total fixation duration, the difference in the results between the medium and high levels of unpredictability was not significant. While differences were evident at the aggregate level, individual preferences may still diverge when the two indicators closed. The user feedback we received during the experiment also raised our doubts about the differences in individual preferences.

“No. 3 (High unpredictability) catches my eye.”

“I like animation No. 3. The fast-paced motion makes me look forward to what will happen in the next second.”

“When I look at the animated logo No. 2 (Medium unpredictability), my eyes are always involuntarily drawn to No. 3, even though at the moment, No. 2 interests me.”

“When I look at No. 3, I feel a little dazzled and don’t know what it wants to express.”

To this end, we conducted local binary comparisons of preference levels with medium to high levels of unpredictability. This hierarchical comparison helps us answer the reasons for the differences in the validation results of H1 between Study 1 and Study 2.

We referred to the calculation of brand preference rate by Peng et al. (2023). Combined with eye-tracking trajectory, respectively counted the preference number of three animated logos. Animated logos with the longest total duration of fixation are recorded as a preference. If the total duration of fixation is close (the difference is less than 15% of the maximum total duration of fixation), the number of fixation and return visits is counted as an auxiliary basis for judgment. Ultimately, the formula for calculating the preference rate of animated logos is: Preference rate = The preference number of animated logos ÷ the total number of preferences.

According to our statistics, except for sample 28, the remaining samples reached the minimum difference threshold. Therefore, a total of 51 samples were ultimately counted, with a preference rate of 2% for animation logos with low unpredictability, 73% for medium unpredictability, and 25% for high unpredictability. The results support the inverted U-shaped effect of perceived unpredictability on sustained attention. When perceived unpredictability increases, users’ sustained attention to animated logos shows a trend of first rising and then falling, which indirectly supports H1.

## 5 General discussion

This study explored the impact of the unpredictability of animated logos on users’ sustained attention through self-reporting and eye-tracking. Incorporate perceived novelty and brand type as mediating and moderating variables to explain the relationship between unpredictability and sustained attention.

### 5.1 Enhanced sustained attention with perceived unpredictability

This study revealed a previously undocumented result: perceived unpredictability is an important factor influencing users’ sustained attention. Study 2 provides further support for the results of Study 1 in an environment with different stimulating materials and different participants. The results confirmed an inverted U-shaped relationship between perceived unpredictability and sustained attention. With the improvement of perceived unpredictability, users’ sustained attention to animated logos shows a trend of rising first and then falling. That indicates that perceived unpredictability is beneficial for enhancing users’ sustained attention. Emerging brands that seek to enhance awareness rapidly should take its importance seriously. However, marketers should be wary of too much perceived unpredictability to prevent it from undermining sustained attention.

Our study also provided some additional interesting findings. Although a maximum threshold for perceived unpredictability was not hypothesized in our study, we observed some consistent patterns in subsequent analyses. Although the overall appeal of animated logos with high perceived unpredictability is not strong, there are still some users who prefer animated logos with higher perceived unpredictability, and most of them are male. However, our study did not examine unpredictability thresholds and gender differences as variables, so these findings should be interpreted with caution. Nevertheless, these findings provide some possibilities for exploring the impact of gender on the highest threshold of perceived unpredictability in the future.

### 5.2 Mediation influence of perceived novelty

This study confirms that perceived unpredictability significantly affects users’ perceived novelty of animated logos and that perceived novelty further affects sustained attention. This discovery supplements previous studies on the impact of perceived novelty, for example, operationalizing ad creativity in B2B advertising (Ferguson et al., 2025), perception of credibility of virtual influencers (Kim et al., 2025), the unconventional layout of online store navigation systems (Wu et al., 2022) and the visual perception influence of packaging design features (Clement et al., 2013), etc. These studies suggest that perceived novelty is an effective means of enhancing sustained attention, and our findings provide additional supporting evidence for this contention.

The mediating effect of perceived novelty explains why users still pay attention to unpredictable animated logos despite their chaotic properties that violate cognitive order. That is an important contribution because designers have found that dazzling, changing images are more likely to attract users’ attention, and these complex animated logos have received a lukewarm response in market practice. Optimizing animation to maximize user attention capture remains a persistent challenge in design practice. Our study highlights hidden ways in which animated logos attract users’ sustained attention. This result provides a basis for setting an optimal threshold of perceived unpredictability. Designers should use perceived novelty as an auxiliary criterion for judging the moderation of perceived unpredictability.

### 5.3 Moderation influence of brand types

As the core representative symbol of brand assets, animated logos aim to maintain the long-term stable operation of brand assets and have an extremely long validity period. From the perspective of brand assets, this study introduces brand types based on the FCB grid as a moderating variable. Informative or Habit Formation are categorized as think brands, corresponding to brand types with utilitarian appeals; Affective or Self-Satisfaction are categorized as feel brands, corresponding to brand types with hedonistic appeals. Previous studies have shown that when a consumer wants to buy think brands, which motive should be cognitively based because of the need for functional performance on one or more readily defined attributes, the utilitarian motive is dominantly activated (McGuire, 1976; Ratchford, 1987). Conversely, when consumers want to purchase feel brands, their motivation is based on emotions because expressive motives (such as self-expression, self-image, ego gratification, and sensory pleasure) are primarily activated (Choi et al., 2012). To be precise, the perception mechanism of think brands is cognitive and typically requires greater cognitive resources for information processing. Users tend to expect feel brand images to be more personalized, figurative, and self-expressive. This expectation aligns with the inherent characteristics of unpredictability, thereby enhancing users’ attention to unpredictable animated logos. A separate comparison of the four quadrants of the FCB grid also reveals that, with the participation of unpredictable animated logos, the Affective and Self-Satisfaction quadrants have an incentive effect on users’ sustained attention. While the Informative and Habit Formation quadrants have an inhibitory effect on users’ sustained attention. This is consistent with the results of the think and feel two-quadrant tests, enhancing the credibility of the results.

### 5.4 Practical significance

Our study is relevant to practitioners in the design and marketing communities because animated logos are increasingly becoming a primary means of brand communication, which represents a huge market for brand investment. Designers are committed to providing brands with visually appealing animated logos, while marketers aim to enhance the brand’s market competitiveness through animated logos. Our research provides practical recommendations for helping animated logos capture users’ sustained attention and bridge the coexistence problem between design and marketing.

The first practical significance of our study is to confirm that the perceived unpredictability of animated logos affects users’ sustained attention. Therefore, practitioners should not underestimate the beneficial effects of perceived unpredictability on brand marketing. The second provides a criterion for practitioners to choose an appropriate level of perceived unpredictability. Put differently, practitioners should utilize the judgment of perceived novelty to limit the adverse effects caused by perceived unpredictability. The third is to remind practitioners to set appropriate animated logos according to brand types and appeals. Complex and unpredictable animated logos are effective for feel brands that pursue trendiness and spiritual enjoyment but are not necessarily suitable for think brands for their meticulousness and excellence. These results have important implications for practitioners who apply perceived unpredictability to enhance users’ sustained attention.

### 5.5 Limitations and prospects

This study suffered from several limitations. First, our participants were mainly college students from South Korea and China, and the sample subjects were relatively young. The advantage compared with the older group, young people are more in line with the user profile of animated logos carried by smart devices. Research on culturally similar groups supports the applicability of this result among young users in Asia. However, it was still unclear whether the evaluations of the unpredictable level of animated logos by other groups (such as different occupations, ages, and genders) were consistent with our results. For example, the older group shows a marked age-related decline in resistance to cognitive interference (Pettigrew and Martin, 2014), so the perceptive unpredictable animations may be more conservative. Gender differences also may be an important controlling factor. Our study discovered differences between men and women in the degree of acceptance of perceived unpredictable levels. Furthermore, all of the participants were from Asia, so the research results may not apply to user groups influenced by other regional cultures. Therefore, future research should consider individual differences and investigate the impact of perceived unpredictability on different user groups.

Second, future research can continue to explore the motion principles in animated logos that affect users’ perceived unpredictability. We defined the beneficial impact of perceived unpredictability on animated logos through 63 practical cases and made a preliminary summary of these motion principles, including speed, amplitude, direction, and structural complexity. However, due to space limitations, we cannot verify the reliability and validity of these motion law factors. Therefore, future research verifies the impact of motion principles on perceived unpredictability through more accurate parameters, thereby advancing the research to practical applications.

Additionally, our original intention is to help marketers enhance the influence of animated logos in marketing. However, our research is still manipulated in an experimental environment, so it is impossible to investigate investigation of the application effect of each animated logo in marketing. Therefore, our research does not include discussions on the brand preference of animated logos. Future studies can move beyond the laboratory and utilize eyeglass eye trackers in market environments to observe market feedback, and use brand preference as an observation indicator to investigate the chain mediating effects of perceived unpredictability, perceived novelty, sustained attention, and brand stickiness. Incorporate market feedback into the observation indicators to further verify our funding.

## Data Availability

The raw data supporting the conclusions of this article will be made available by the authors without undue reservation.
