# Supplementary material for: Embracing chaos: the unpredictability of animated logos shapes users’ sustained attention
Source: Front Psychol. 2025 Aug 6;16:1642722. doi: 10.3389/fpsyg.2025.1642722 (PMC12364914; doi:10.3389/fpsyg.2025.1642722)
Supplement: Supplementary file 1 [file Presentation_1.pdf]

Supplementary Material

1 Experimental Materials for Study 1

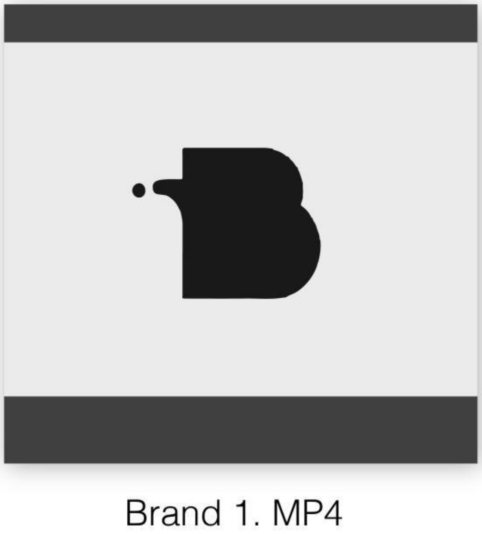

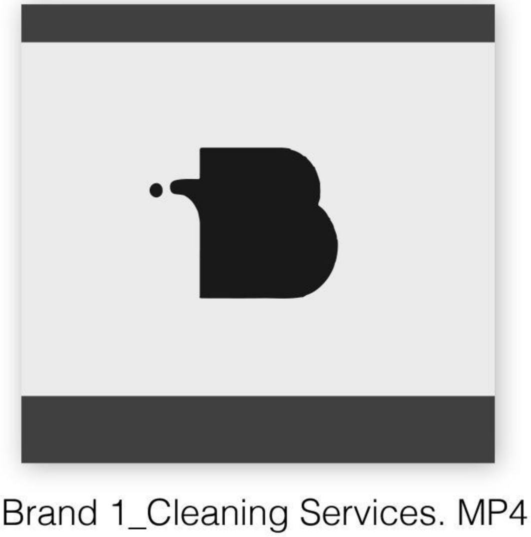

Supplementary Figure 1. Control group.

Supplementary Figure 2. Experimental group.

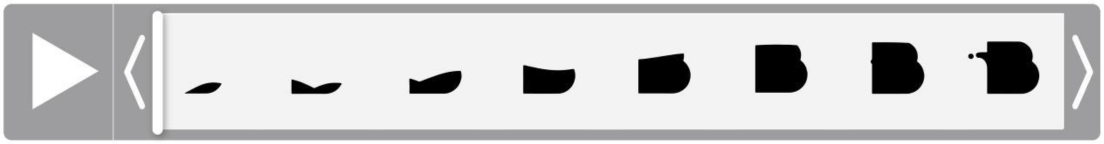

Supplementary Figure 3. Schematic of the control group's video material.

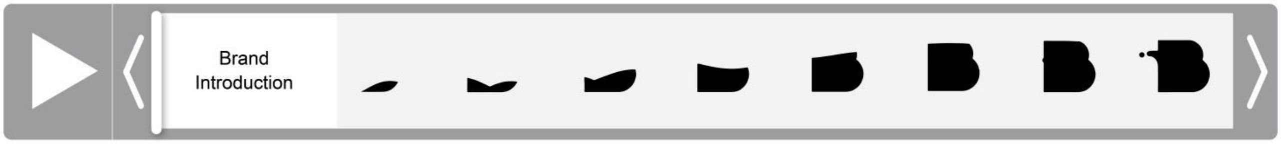

Supplementary Figure 4. Schematic of the experimental group's video material.

To analyze the motion principles affecting perceived unpredictability in animated logos. We counted the degree of perceived unpredictability (questionnaire items 7-10) of the 63 animated logos based on the results. We ranked them in descending order (see Supplementary Table 1).

Supplementary Table 1. Ranking of unpredictability of experimental materials in Study 1.

Ranking	Material ID	Mean	SD	N	Ranking	Material ID	Mean	SD	N
1	No. 53	5.82	0.19	36	33	No. 26	4.56	0.25	26
2	No. 46	5.61	0.22	28	34	No. 54	4.54	0.30	24
3	No. 3	5.54	0.10	42	35	No. 33	4.52	0.23	31
4	No. 16	5.53	0.20	29	36	No. 35	4.51	0.23	31
5	No. 32	5.52	0.16	40	37	No. 12	4.50	0.20	34
6	No. 31	5.50	0.23	28	38	No. 63	4.41	0.21	25
7	No. 30	5.49	0.17	38	39	No. 58	4.35	0.35	21
8	No. 10	5.48	0.17	30	40	No. 42	4.29	0.22	26
9	No. 49	5.41	0.25	24	41	No. 13	4.26	0.23	33
10	No. 29	5.27	0.18	31	42	No. 28	4.21	0.20	34
11	No. 43	5.26	0.20	35	43	No. 60	4.20	0.23	21
12	No. 11	5.25	0.18	27	44	No. 23	4.16	0.18	35
13	No. 34	5.21	0.21	33	45	No. 61	4.07	0.23	19
14	No. 39	5.20	0.22	29	46	No. 22	3.96	0.27	28
15	No. 8	5.15	0.23	31	47	No. 19	3.95	0.26	23
16	No. 9	4.98	0.22	31	48	No. 27	3.83	0.15	34
17	No. 55	4.96	0.27	20	49	No. 18	3.78	0.20	29
18	No. 17	4.90	0.24	21	50	No. 38	3.76	0.17	32
19	No. 4	4.88	0.28	31	51	No. 21	3.64	0.17	35
20	No. 25	4.86	0.28	29	52	No. 44	3.55	0.22	20
21	No. 62	4.83	0.24	21	53	No. 37	3.42	0.23	36
22	No. 57	4.78	0.31	24	54	No. 48	3.33	0.20	21
23	No. 15	4.77	0.14	40	55	No. 47	3.33	0.17	22
24	No. 36	4.76	0.12	27	56	No. 1	3.30	0.16	43
25	No. 45	4.75	0.27	25	57	No. 7	3.23	0.24	37
26	No. 2	4.73	0.26	31	58	No. 50	3.23	0.21	33
27	No. 56	4.72	0.23	23	59	No. 40	2.87	0.21	31
28	No. 59	4.69	0.14	24	60	No. 24	2.71	0.16	34
29	No. 41	4.66	0.24	32	61	No. 14	2.22	0.20	32
30	No. 6	4.65	0.29	25	62	No. 51	2.19	0.16	27
31	No. 5	4.57	0.17	36	63	No. 20	2.04	0.15	25
32	No. 52	4.56	0.20	21					

We manipulated materials in Study 2 by using perceived unpredictability as the only variable. To manipulate perceived unpredictability, we needed to specify the motion principles that influence the degree of perceived unpredictability. Based on the results of the assessment of the unpredictability of the stimulus material in Study 1, we selected five videos from each of the pre-ranking and post-ranking and analyzed their motion principles inductively. After disassembling the video frame by frame through Adobe After Effect, we found that speed, amplitude, direction, and structural complexity comprehensively affect the perceived unpredictability of animated logos.

First, stimulus materials with high perceived unpredictability mostly exhibit the characteristic of rapid movement. For instance, Material 3 (Mean = 5.54, High perceived unpredictability), from 00:03.11 to 00:03.47, the graphic rotated 1,080 degrees over 36 milliseconds. Conversely, in Materials 24 (Mean=2.71, Low perceived unpredictability), from 00:01.49 to 00:03.44, it takes 115 milliseconds for the figure to rotate 360 degrees, and the movement speed is extremely slow.

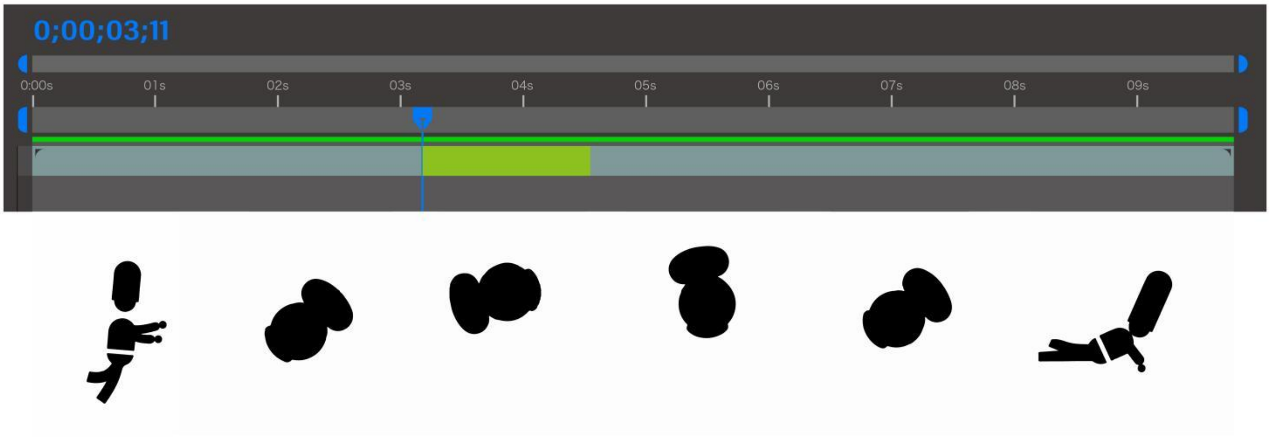

Supplementary Figure 5. Schematic of Materials 3 (Mean = 5.54, High perceived unpredictability).

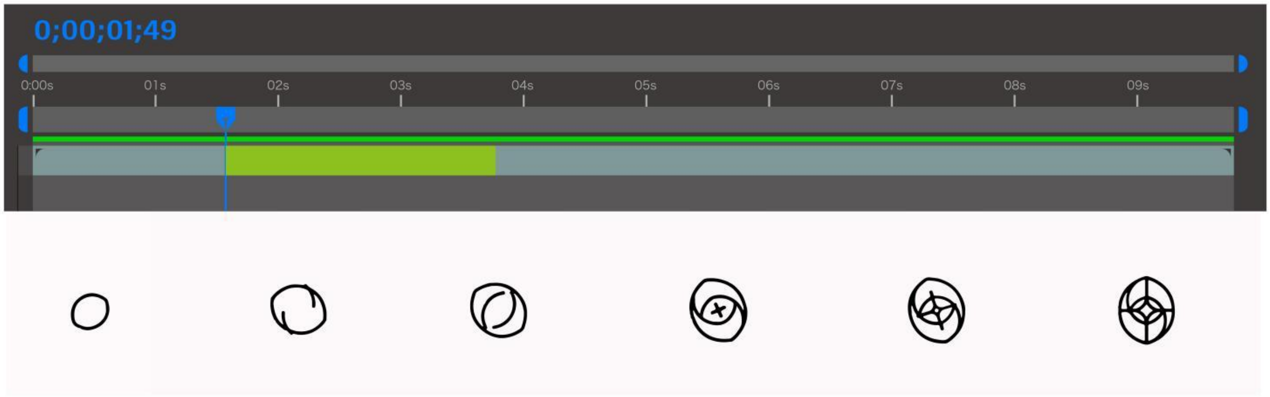

Supplementary Figure 6. Schematic of Materials 24 (Mean=2.71, Low perceived unpredictability).

Second, the graphics with high perceived unpredictability have a larger amplitude. In Material 14 (Mean = 2.22, Low perceived unpredictability), the character is simulating the action of swimming with only the limbs showing significant movement while the rest of the body remains stationary. In contrast, the larger amplitude in Material 16 (Mean = 5.53, High perceived unpredictability) gives the whole image dramatic tension.

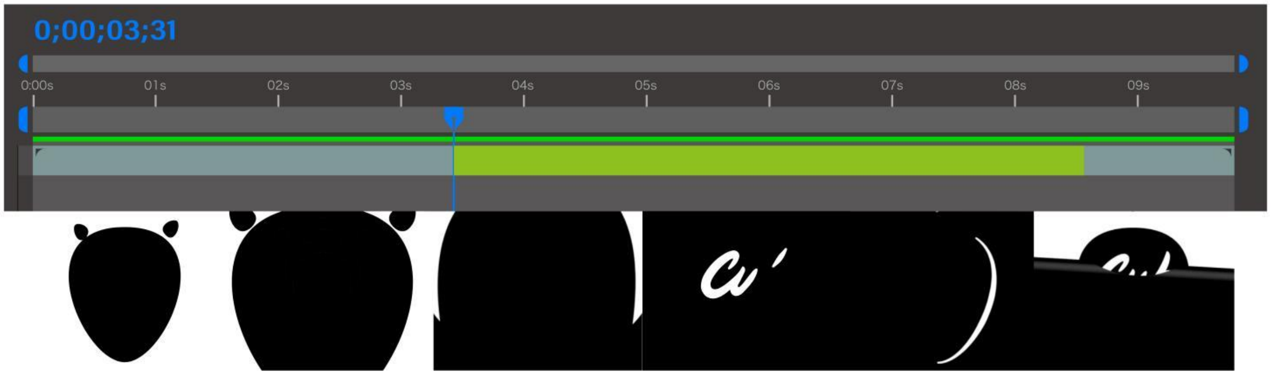

Supplementary Figure 7. Schematic of Materials 16 (Mean = 5.53, High perceived unpredictability).

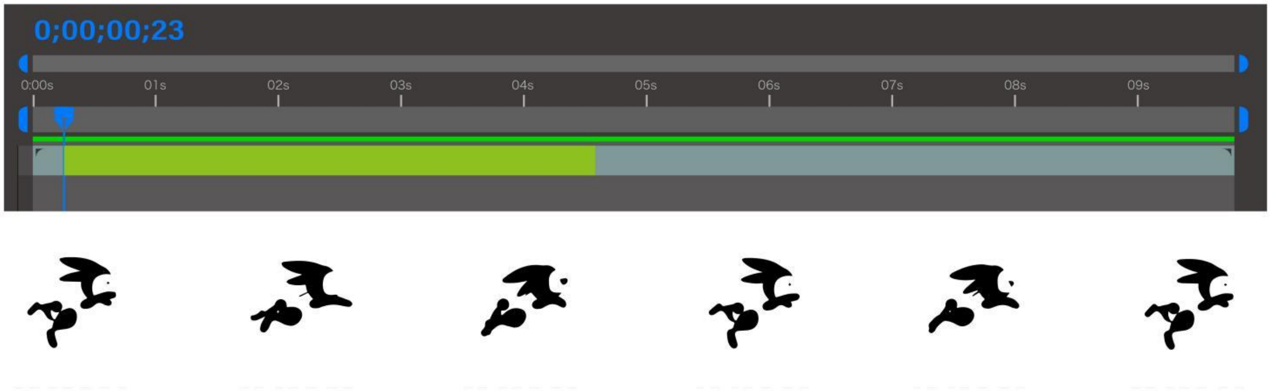

Supplementary Figure 8. Schematic of Materials 14 (Mean = 2.22, Low perceived unpredictability).

Third, animations of high perceived unpredictability have a more complex direction. For example, in Materials 53 (Mean = 5.82, High perceived unpredictability), multiple directions such as jumping (upward), panning (left and right), rotating (around the center), free-falling (downward), and aspect (zooming in and out). Whereas the graph in Materials 51 (Mean = 2.19, Low perceived unpredictability) shows only horizontal translation.

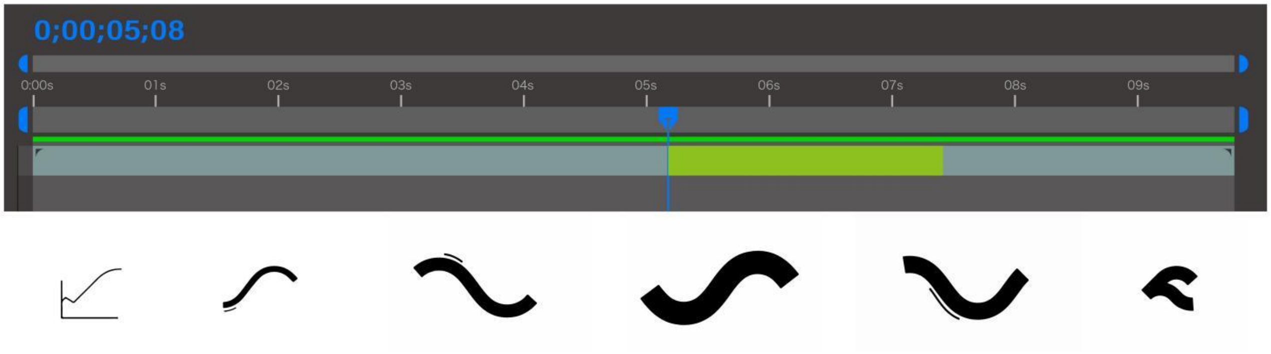

Supplementary Figure 9. Schematic of Materials 53(Mean = 5.82, High perceived unpredictability).

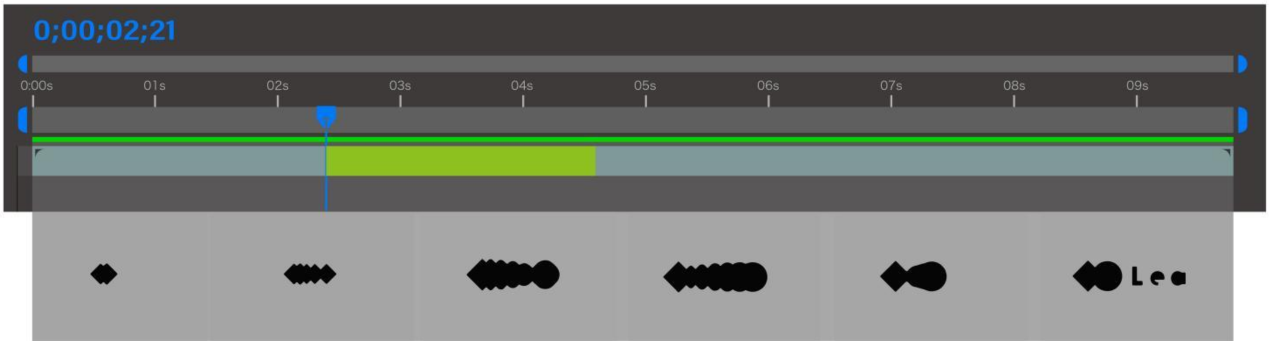

Supplementary Figure 10. Schematic of Materials 51(Mean = 2.19, Low perceived unpredictability).

Finally, structural complexity is also responsible for increasing the perceived unpredictability. From 00:03.36 to 00:05.27 of Material 46 (Mean = 5.61, High perceived unpredictability), the number of major animations exceeds 8. In contrast, Materials 20 (Mean = 2.04, Low perceived unpredictability) demonstrated repetitive motion of only one set of animations.

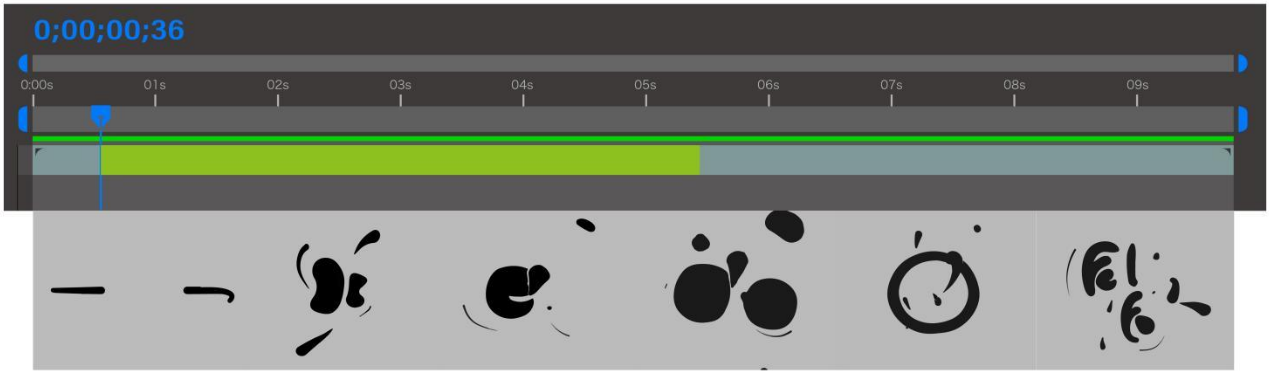

Supplementary Figure 11. Schematic of Materials 46(Mean=5.61, High perceived unpredictability).

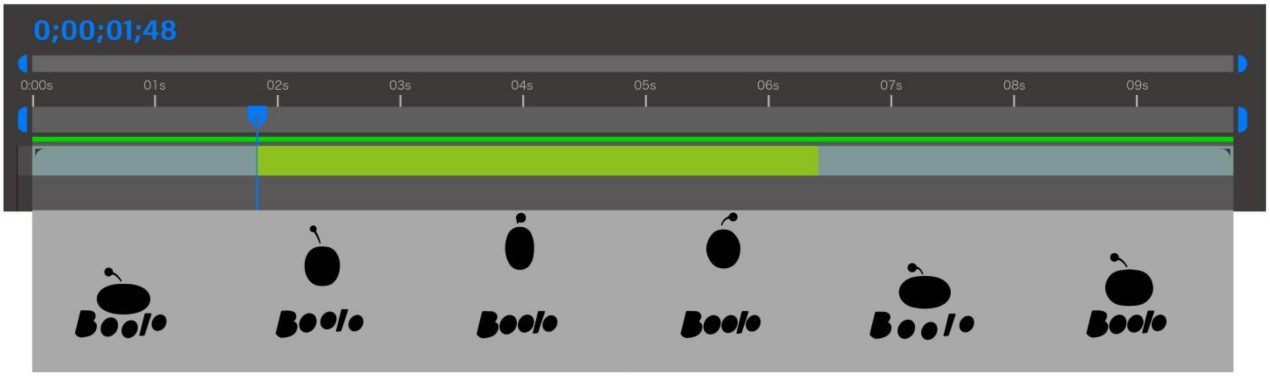

Supplementary Figure 12. Schematic of Materials 46(Mean=5.61, High perceived unpredictability).

Through analysis, we find that animated logos with faster speed, greater amplitude, more directions, and more complex structures usually exhibit a higher level of perceived unpredictability. Therefore, in Study 2, we manipulated the stimulating materials based on the above motion principles to make them exhibit obvious and unpredictable differences.

2 Experimental Materials for Study 2

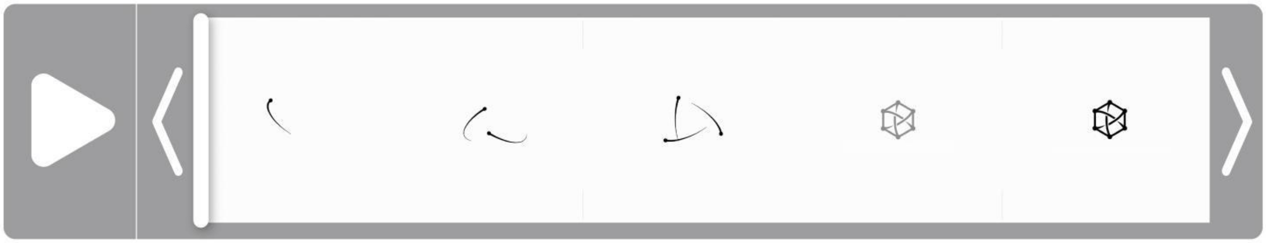

Supplementary Figure 13. Schematic of animated logo with low perceived unpredictability.

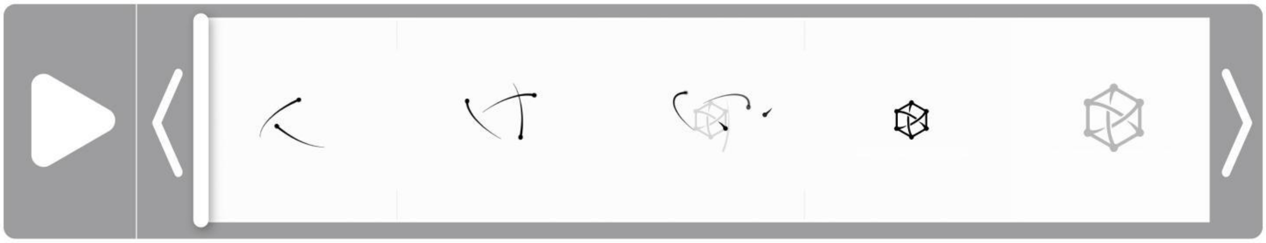

Supplementary Figure 14. Schematic of animated logo with medium perceived unpredictability.

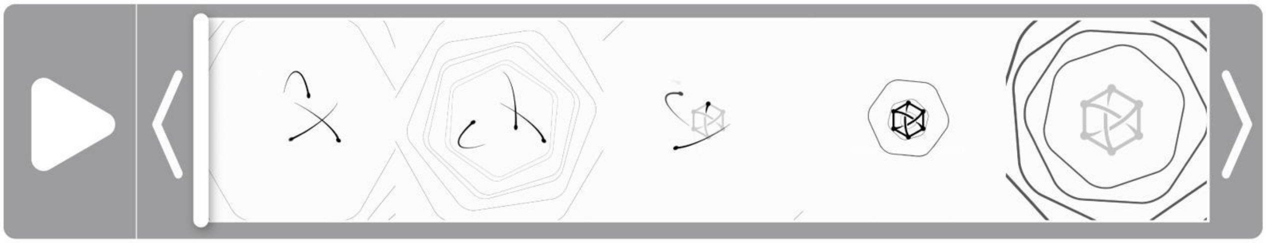

Supplementary Figure 15. Schematic of animated logo with high perceived unpredictability.

To obtain video material in MP4, please contact the relevant author.
